# Modelling agricultural land abandonment in a fine spatial resolution multi-level land-use model: An application for the EU

**DOI:** 10.1016/j.envsoft.2020.104946

**Published:** 2021-02

**Authors:** Carolina Perpiña Castillo, Chris Jacobs-Crisioni, Vasco Diogo, Carlo Lavalle

**Affiliations:** a European Commission, Joint Research Centre (JRC), Ispra, Italy; b Swiss Federal Research Institute WSL, Zürcherstrasse 111, Birmensdorf, CH-8903, Switzerland

**Keywords:** Agricultural land abandonment, Territorial modelling, EU Reference scenario, European risk map, Validation

## Abstract

In the majority of EU Member States, agricultural land is expected to decrease not only due to land-use changes in favour of urban expansion and afforestation but also to land abandonment processes. The knowledge on location and extent of agricultural land abandonment is relevant for estimating local external effects and adapting policy interventions. Currently, multi-level land-use models are able to capture determined processes of demand-driven redevelopment. However, land abandonment is much more difficult to capture because of its more ambiguous definition and the lack of data on its spatial distribution. This paper presents a method to explicitly model agricultural abandonment as a choice of disinvestment, which in turn is embedded in a utility-based land-use modelling framework that projects land-use changes for the EU and the UK. Validation exercises using observed spatial distribution of abandoned farmland show that the proposed method allows to model abandonment with acceptable accuracy.

## Highlights

• LUISA is a modelling platform able to dynamically project agricultural land abandonment.
• Biophysical, agricultural socio-economic and regional factors are key drivers to estimate abandonment risk.
• The model outcomes project more than 5.6 M ha of abandoned land by 2030 in the EU and the UK.
• Risk areas for land abandonment are widespread in the EU countries and not limited to mountain regions.
• We present a long-term territorial assessment that might guide different strategies for preventing land abandonment.
• Data availability and resolution are key elements for modelling and validating farmland abandonment.

## 1 Introduction

In Europe, the abandonment of agricultural lands has been an important land-use change process at least since the 19th century (Mather, 2001). It still is a topical issue, reflecting the post-war and post-Soviet trends of rural depopulation and loss of competitive advantages in the rural economy (Baldock et al., 1996; Prishchepov et al., 2012), and is particularly problematic in mountainous, remote and semiarid areas (MacDonald et al., 2000; Benayas et al., 2007). A problem that is pervasive in the land abandonment literature is the difficulty of defining, identifying and observing the process at hand. Indeed, agricultural land abandonment can be defined in many different ways^1^; but commonly refers to land that was previously used to grow crops or for grazing, does not have farming functions anymore (i.e. a total cessation of agricultural activities); and has not been converted to forest or artificial areas either (Hart et al., 2013; Pointereau et al., 2008; FAO, 2006). Many factors are involved in this complex and multi-dimensional phenomenon that is primarily triggered by low productivity and land degradation and occurs, more often in remote and mountainous regions, with soil or climate conditions that are unfavourable for agriculture. Secondary drivers such as rural depopulation, socio-economic factors, policies or inefficient farm structure can further accelerate land abandonment (Van der Zanden et al., 2017; Lasanta et al., 2016).

The abandonment of agricultural land can cause undesirable environmental, socio-economic and landscape impacts; for instance, biodiversity loss, landscape homogenization, increased fire risk, soil erosion and soil degradation, as well as increase of the area of agriculture intensification (Keenleyside and Tucker, 2010; Hart et al., 2013; Lasanta et al., 2016). However, agricultural abandonment does not only entail a higher pressure on biodiversity and natural resources, but also causes decline of local agricultural incomes and employment, and is thus directly linked to population dynamics. Especially in mountainous or remote rural areas the loss of agricultural income aggravates often already weak economic and social structures (European Union, 2013). In addition, agricultural land abandonment is linked to the loss of local agricultural practices and knowledge (Gellrich et al., 2007; Baldock et al., 1996).

On the other hand, where agricultural abandonment coincides with favourable climate and soil conditions it can lead to environmentally valuable natural succession. This re-vegetation process may entail important positive benefits such as improving soil organic matter content, stabilisation of soils, carbon sequestration, regulation of water flow, and habitat restoration with an improvement in species number and biodiversity (Corbelle-Rico and Crecente-Maseda, 2008; Estel et al., 2015). All in all, the trade-offs between positive or negative impacts that can result from abandoning agricultural land are largely site-specific, depending on the geographic location and related biophysical conditions, cultural heritage, environmental, political and socio-economic preferences (Hart et al., 2013; Verburg et al., 2009).

In many countries in Europe, the potentially negative impacts of agricultural abandonment have been addressed via a broader set of policy instruments that aim to alleviate the negative consequences or even reverse abandonment trends in its early stages. For decades the European Union (EU) has been intervening in agricultural markets with specific payment schemes dedicated to marginal farming areas (Eliasson et al., 2010; Keenleyside and Tucker, 2010; Koomen et al., 2008). The “Less Favoured Areas” (LFA) and, more recently, the “Areas facing Natural or other specific Constraints” (ANC) support schemes, have been providing compensation to farmers who continue to farm despite unfavourable conditions (EC, 2005; EU, 2013). In addition, supportive national legislations are crucial to tackle the land abandonment problem, as national legislators are typically more informed on local characteristics and needs (Corbelle-Rico and Crecente-Maseda, 2008).

During the last decades, considerable effort has been put in mapping, quantifying and assessing the major impacts and drivers that entail abandonment land processes. Some studies on land abandonment focused on concepts and drivers, highlighting the importance of environmental, socio-economic and farm management factors (Corbelle-Rico and Crecente-Maseda, 2008; FAO, 2006; Benayas et al., 2007; García-Ruiz and Lana-Renault, 2011; Keenleyside and Tucker, 2010, Terres et al., 2015; Lasanta et al., 2016), while others have been centred on the efficiency of policy measures (MacDonald et al., 2000; Walford, 2002; Renwick et al., 2013 Corbelle-Rico and Crecente-Maseda, 2014). Positive and negative consequences of land abandonment, as already mentioned, are another recurrent topic (Baldock et al., 1996; MacDonald et al., 2000; Van der Zanden et al., 2017; Nainggolan et al., 2012), often applied to case studies located in Mediterranean Europe^2^ and mountainous areas (Falcucci et al., 2007; Poyatos et al., 2003; Etienne et al., 2003; Lasanta et al., 2016; MacDonald et al., 2000; Corbelle-Rico et al., 2012). Recently, other studies have used remote sensing (Estel et al., 2018; Alcantara et al., 2013) to distinguish productive, fallow, and recultivated farmland. Those studies have done so at European and global scales respectively, by calculating NDVI time series from different satellite sensors at high spatial resolution.

Given the political relevance of agricultural abandonment in Europe, an estimate of where and how much abandonment will happen in the future would be useful. Unfortunately, so far less attention has been given to modelling techniques to obtain estimates of future abandonment locations, typically developed in existing spatially dynamic modelling systems. Previous studies built different scenarios in order to explore possible future developments and impacts, and were based mostly on econometric techniques (Nowicki et al., 2006; Westhoek et al., 2006; Verburg and Overmars, 2009; Meiyappan et al., 2014; Price et al., 2015; Van der Zanden et al., 2017). These existing models mainly attempt to analyse trends and changes in landscape and spatial patterns over time, but are relatively limited with regard to the representation of the agricultural land abandonment process (Keenleyside and Tucker, 2010). Other location models for farmland abandonment are based on the assumption that abandonment likely occurs where local suitability for agricultural practices is relatively low (Verburg and Overmars, 2009; Meiyappan et al., 2014). Recent works suggest, however, that marginalization of agriculture is not only driven by poor biophysical characteristics or lack of demand for produce. Abandonment of agricultural land as an economic resource typically occurs when it has ceased to generate sufficient income flows, and the available options (within the restraints of farmers' knowledge and capacities) for adjusting resource use, farming practices or farm structure have been exhausted (MacDonald et al., 2000). Thus, other structural and monetary factors that affect farmers' income and abilities also play a decisive role; for instance, the farmers’ age and qualification, existing subsidy schemes and differential competitive advantages among rural regions.

A more refined method to model locations of farmland abandonment is called for. The paper at hand presents a method to explicitly model future local agricultural abandonment processes as a result of economic decisions on the use of land, within an integrative, spatially dynamic land-use modelling framework. The implementation of the method is illustrated with the LUISA Territorial Modelling Platform, a model that dynamically simulates population, land-use and accessibility changes across the EU and the UK (The United Kingdom) at a 100 m resolution in order to assess local and cross-policy externalities. Future land-use trends and major drivers of land abandonment are simulated under the EU Territorial Reference Scenario 2017 (Jacobs-Crisioni et al., 2017). The territorial assessment of agricultural abandonment trends and its associated impacts is presented at national, regional (NUTS3) and grid level for all EU countries and the UK up to 2030. In addition, a method to quantify land-use/cover flows is used to represent the main transitions between land uses that are simulated. In particular, this paper presents aggregated land conversions that, according to the introduced model procedure, supersede agricultural abandonment (Perpiña Castillo et al., 2018, Perpiña Castillo et al., 2019).

The rest of this paper is structured as follows. Firstly, we briefly introduce the general land-use modelling framework of LUISA (Section 2.1). Then, we further describe the method for deriving the European risk map of agricultural land abandonment (Section 2.2). Section 2.3 describes agricultural abandonment as part of a utility-based land-use modelling framework as well as outlines the future projections for agricultural land abandonment up to 2030. Section 2.4 describes the method for validating the proposed modelling approach. In section 3 the main outcomes of the study are presented and analysed, and finally, section 4, 5 summarises and discusses the main points of the proposed method versus other studies.

## 2 Material and methods

Within the LUISA modelling framework, land abandonment is thus conceptualised as a temporary phenomenon that may happening even without demand reduction, as a consequence of a transition of the agricultural production system towards an optimal spatial distribution (section 2.1, 2.2). A combination of factors is assumed to be involved in agricultural land abandonment. Land-use competition, biophysical conditions, agricultural economics, farm structure, demographic and geographical factors are all expected to play a role (section 2.3.1, 2.3.2, 2.3.3). These individual factors are first represented in maps using a variety of data sources, and then integrated together to build a composite map of agricultural land abandonment risk for the whole EU and the UK at a fine resolution from 2015 to 2030 (section 2.3.4). This risk map is then used within the LUISA model as a compound local driver for simulating agricultural abandonment processes, given a set of regional demands for land-based functions and activities, as it is explained in the next section.

### 2.1 LUISA territorial modelling platform: agricultural land and its abandonment

LUISA is a pan-European modelling platform^3^ that provides alternative scenarios of territorial development, in order to understand the local impacts and externalities of EU trends and policies. The current configuration, the EU Territorial Reference Scenario 2017, integrates the most recent and accurate information available, including past and future time series of socio-economic and environmental aspects. It also accounts for existing European policies and legislation (e.g. Common Agricultural Policy, Renewable energies, Trans-European Transport Network, EU Biodiversity strategies and protection of Natura 2000 areas). For a more detailed account on LUISA modelling framework and data sources, we refer to Jacobs-Crisioni et al. (2017). For a comprehensive description of LUISA's territorial reference scenario 2017, see Appendix A.

Discrete land-use changes in LUISA are modelled by optimizing the expected local utility values for land uses. Optimization is constrained by the available land in a region and by input expectations on total land area in the region that is needed by the modelled land uses. A key input thus considers projected regional demand for agricultural land. These demand projections are obtained from the 2016 CAPRI baseline^4^, which integrates main policy, macro-economic and market assumptions up to 2030, while being consistent with the EU Agricultural Outlook 2016–2026 (European Commission, 2016). Agricultural demand is imposed in LUISA as land area required for the expected production of food, feed and energy crops, and is expressed through a number of agricultural land classes that are aggregations of CAPRI commodities. The following systems are identified: arable farming (including rice), livestock/grazing systems, mixed crop-livestock production, permanent crops and bioenergy crops (Jacobs-Crisioni et al., 2017; Perpiña Castillo et al., 2016).

Given regional demand for agricultural land and other land uses, a dedicated discrete allocation mechanism available in the open-source GeoDMS software (2019) iteratively adapts the local utility until land-use distributions are found that satisfy the modelling constraints (Hilferink and Rietveld, 1999). The underlying assumption is that grid cells function as implicit agents, who change the use of their land if opportune in terms of utility and regional demand, choosing from the bounded set of land-use options that are modelled.

Land-based functions require investments with a long-term time horizon. Utility is, therefore, computed as the net present value (NPV)^5^ of that land cover at a specific location. To be regarded as economically attractive, an investment should have a strictly non-negative NPV. For all land uses, utility is estimated in a spatially-explicit way given local and global parameters, similarly to the approach proposed by Koomen et al. (2015) and Diogo et al. (2015):

(1) $$NPV_{r}=-I+\overset{n}{\underset{t=y}{\sum }}\frac{R_{r,t}-C_{t}}{{\left(1+d\right)}^{t-y}}$$

where I are the initial investment costs (in €/ha, e.g. land clearing/demolition costs, building costs, acquiring agricultural machinery); $R_{r,t}$ are the annual gross revenues for raster cell r in year t (in €/ha, obtained from e.g. rental income, revenues from selling crops, subsidies); $C_{t}$ are annual costs (in €/ha, e.g. maintenance costs, field operations in agriculture); n is the investment time-horizon (in years); d is the discount rate.

The time horizon, annual costs and discount rates are held fixed in the model regardless of location and modelling time. Initial investment costs do depend on the existing land cover in a specific location, as the existing physical make up of a location may call for clearing or demolition operations. Revenues are highly dependent on location, being calculated as follows:

(2) $$R_{r,t}=S_{r,t}\text{*}\max R_{t}$$

where $S_{r,t}$ the local suitability, i.e. the percentage of maximum revenue to be obtained from a specific land-use at a given location, defined as the probability that a particular land cover exists given a set of geographic variable values, and estimated through binomial logistic regression analyses on observed land-cover patterns per country; $\max R_{t}$ is the maximum revenues (in €/ha), i.e. the annual revenues that are assumed to be obtained from a particular land-use in case the local suitability is optimal (i.e. $S_{r,t}$ = 100%).

The computed NPVs are implemented in the allocation algorithm, by employing a logit-type approach derived from discrete-choice theory (McFadden, 1978). Discrete choice theory aims to explain and predict the outcome of decision-making process of economic agents when choosing among mutually exclusive alternatives. The discrete choice model assigns probabilities for the different alternatives according to the utility of those alternatives in relation to the total utility of all alternatives. When applying this model in a spatially-explicit way, the probability of choosing among mutually exclusive land-based activities in a given location is computed as follows:

(3) $$X_{r,i}=\frac{e^{\beta +U_{r,i}}}{\sum_{k=1}^{K}e^{\beta +U_{r,k}}}$$

where $X_{r,i}$ is the probability of alternative land-use i being chosen in raster cell $r;\;U_{r,i}$ is the utility of alternative i in raster cell r (i.e. the NPV of that activity in that particular location); $U_{k,i}$is the utility of alternative k in raster cell r; K is a finite number of mutually exclusive alternatives for land-based activities, and β is a parameter to adjust the model sensitivity (typically 1 as default value).

### 2.2 Modelling future agricultural land abandonment

In LUISA, the extent, location and timing of farmland abandonment is modelled in three separate classes, namely through abandoned arable crops, permanent crops and fields used for livestock. To do so, both local likeliness and regional expectations of abandonment need to be provided for every 5-years model step. Expectations on future regional agricultural abandonment are dynamically quantified for each modelled country separately, based on expected shares of land abandonment. Those were quantified based on per-annum percentual losses of Utilized Agricultural Area (UAA) as observed in Corine Land Cover between 2000 and 2012, and are further supported by the reference values taken from the modelling exercises presented in Van der Zanden et al. (2017). In every modelling time step, percentage loss is converted into an absolute expected loss of area using prior total agricultural area and assigned a maximum and minimum value range, in order to have sufficient degrees of freedom for the model to find an optimal solution.

At the local level, abandonment is simulated in LUISA's utility optimization approach as an alternative choice available to all grid cells that are currently used as agricultural land. This approach, thus, considers abandonment a separate disinvestment decision that may be the highest utility outcome in specific locations and contexts. So-called allow rules govern which transitions between land uses are permitted within the simulation. They are imputed in the model by imposing that the NPV values for a non-allowed transition are below the minimum threshold of the discrete allocation method, so that, effectively, disallowed transitions are not considered. Through such allow rules, only agricultural land types can become abandoned agricultural land while previously abandoned agricultural land can be converted into any land-use type (residential, forestry, etc.), save other abandoned land classes, in a subsequent time step.

Agricultural revenue and cost estimates were obtained from Ustaoglu et al. (2016). Abandonment is modelled through assuming zero cost and a small fraction of the agricultural revenue to proxy revenues from disinvestment. As a comprehensive EU-wide map of agricultural abandonment is unavailable, similar functions could not be induced for abandonment probability from empirically observed land-use patterns. An agricultural abandonment risk map has, therefore, been deduced by quantifying and mapping relationships found in previous studies (see next section 2.3.).

### 2.3 European risk map of agricultural land abandonment

The risk map^6^ of agricultural land abandonment is created by combining many factors into three groups, related to biophysical, agricultural socio-economical, and demographic and geographic factors (Table 1). These factor groups are defined by adapting and combining several methods from the recent literature (Benayas et al., 2007; Pointereau et al., 2008; Confalonieri et al., 2014; Terres et al., 2015; Lasanta et al., 2016; Levers et al., 2018). Each factor corresponds to a spatial thematic layer or statistical information at regional level from different data sources (see Appendix B, Table B.1, Table B.2 and Table B.3). The factor groups are further detailed in the next sections.

**Table 1:** Main factors that drive agricultural land abandonment.^a^

Biophysical factors	Agriculture's socio-economic and farm structure factors	Demographic and geographic factors
Length of growing period	Age of farmers	Population density
Soil Organic matter	Farmer qualification	Remote areas
Soil texture	Farm size	
Root depth	Rent paid	
Soil pH	Rented UAA	
Salinity and sodic	Farm income	
Precipitation	Farm investment	
Soil drainage	Farm scheme (subsidies)	
Slope		

a The rationale behind the selection of these factors that drive agricultural land abandonment as well as the cut-off values to be classified as severe natural conditions can be found, for instance, in Eliasson et al. (2010); Confalonieri et al., 2014; European Union, 2013. See Appendix B, Appendix C for detailed information about each factor.

#### 2.3.1 Biophysical factors

**Fig. C.1:**
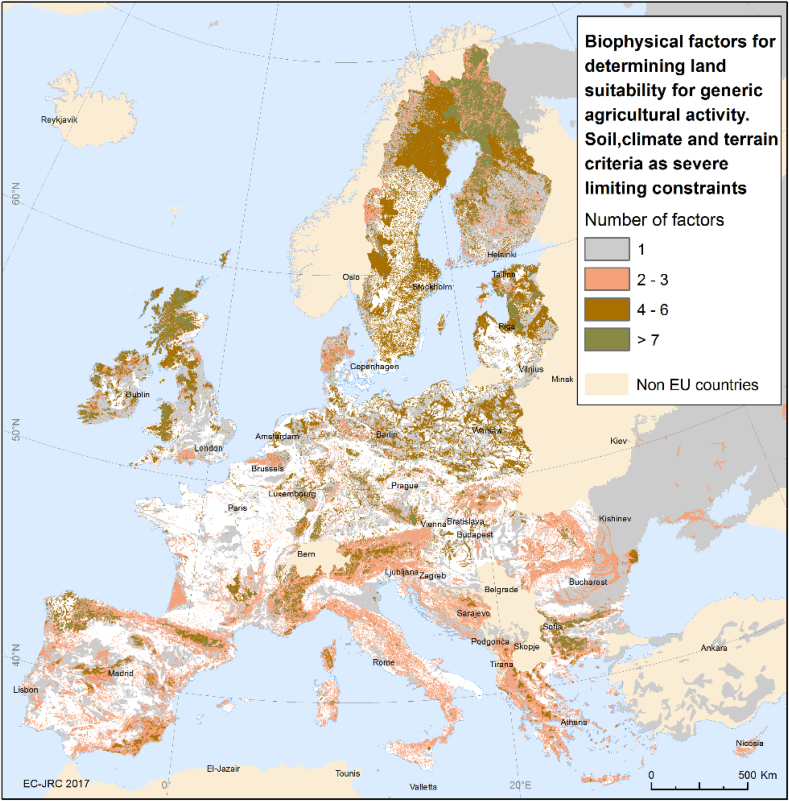
Number of biophysical factors spatially concurring for classifying land suitability for agricultural generic activities in the EU countries and the UK.

A set of nine factors, dealing with soil, climate and terrain criteria^7^, is selected to determine where constraining natural conditions occur, reflecting guidelines from EU Regulation No 1305/2013 (European Union, 2013; Eliasson et al., 2010), Annex III “Biophysical criteria for delimitation of areas facing natural constraints”. For generic agricultural activities, the selected constraining conditions are expected to increase the risk of land abandonment. This is spatially represented by merging the nine factors as a composite map of biophysical risk of abandonment (Appendix C, Fig. C.1).

The selection of these criteria is supported by many studies. As described in Alonso-Sarria et al. (2016) and Corbelle-Rico and Crecente-Maseda (2014) variables such as slope, precipitation and irrigated areas are relevant variables for abandonment. In fact, water availability is an important factor linked directly with agricultural profitability, where even irrigated plots partially rely on precipitation. Land use is considered an important variable in relation with land abandonment in the sense that rainfed crops are more prone to abandonment than irrigated crops (García-Ruiz, 2010; Nadal-Romero et al., 2016). In relation to soil properties, soils characterized by low nutrient content, high salinity, high proportion of clay and shallow soils are more prone to abandonment (Alonso-Sarría et al., 2016; Romero-Díaz et al., 2017). Negative multiplication effects among constraining conditions are also taken into account, as proposed by Terres et al. (2014), although in a simplified way. Here, locations where at least two severe limiting conditions coincide are considered to suffer severe limitations for agricultural activity, thus having higher abandonment risk. The values of these variables are held fixed throughout the simulation period.

#### 2.3.2 Socio-economic and farm structure factors

**Fig. C.2:**
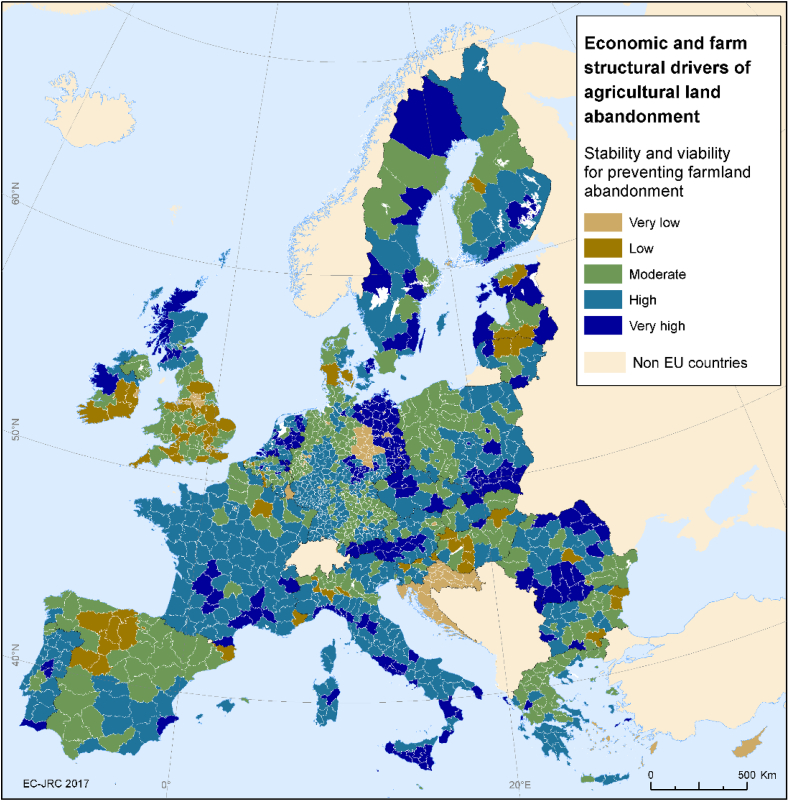
Map combining economic and farm structure drivers of agricultural land abandonment in the EU countries and the UK, period 2005–2010

**Fig. C.3:**
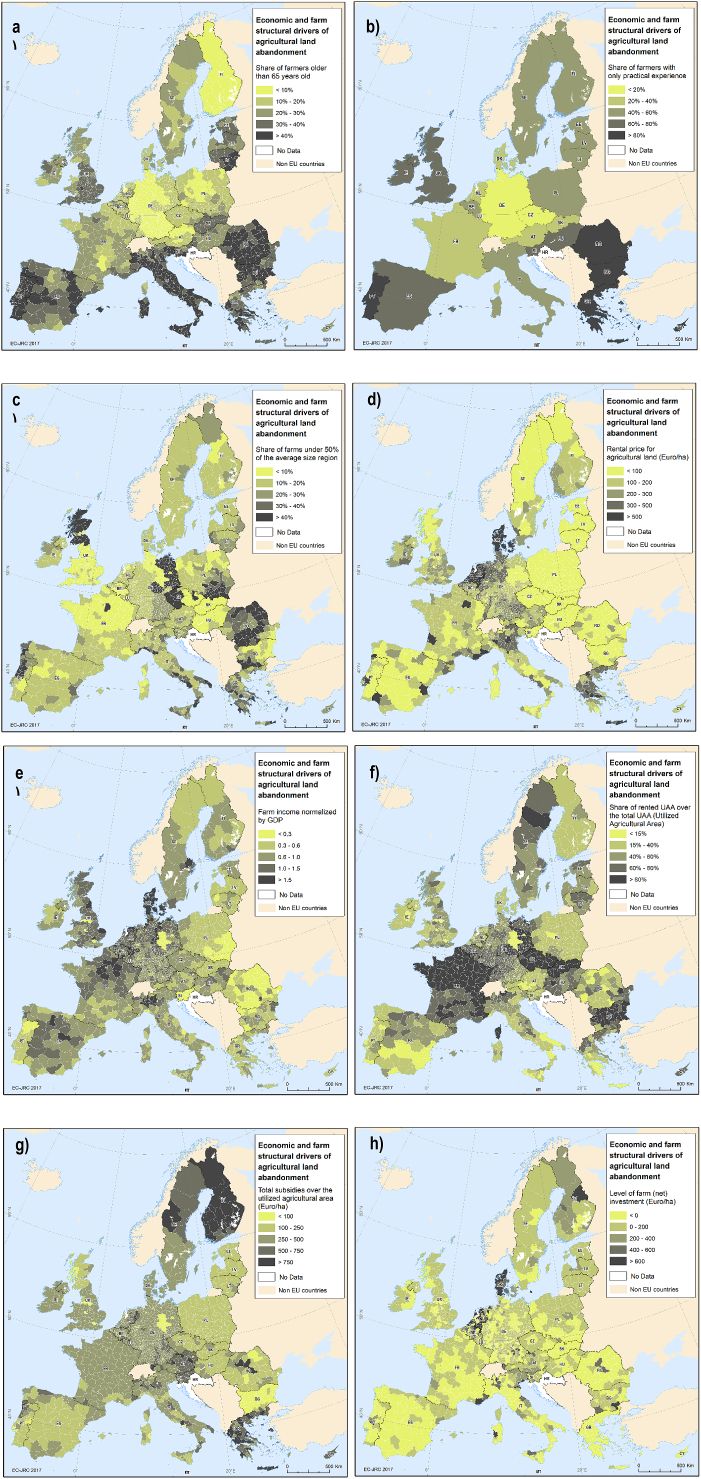
Individual economic and farm structural factors for determining the stability and viability of farming activities for preventing agricultural land abandonment in the EU countries and the UK. a) Share of farmers older than 65 years old, b) Share of farmer with only practical experience, c) Share of farms under 50% of the average size region, d) Rental price, e) Farm income, f) Share of rented UAA, g) Total subsidies and h) Farm investment.

Economic and farm-structure agricultural data is used to represent the stability, viability and performance of regional agricultural systems, indicating resilience against farmland abandonment. These datasets are mainly gathered from FADN^8^ (Farm Accountancy Data Network) and DG EUROSTAT-FSS^9^ (Farm Structure Survey). A harmonization exercise^10^ is necessary to merge FADN and FSS data in a complete and consistent database. The values of all variables selected here are averaged over the period 2005–2010 and subsequently considered static throughout the simulation period. A normalization process is applied to facilitate comparison of results between countries. Table B.2 (Appendix B) shows the main characteristics (description and data source) of the eight factors involved. Figure C.2 (Appendix C) shows the spatial combination of economic and farm structure factors, while in Figure C.3 (Appendix C) each factor is mapped individually.

#### 2.3.3 Demographic and geographic regional factors

**Fig. C.4:**
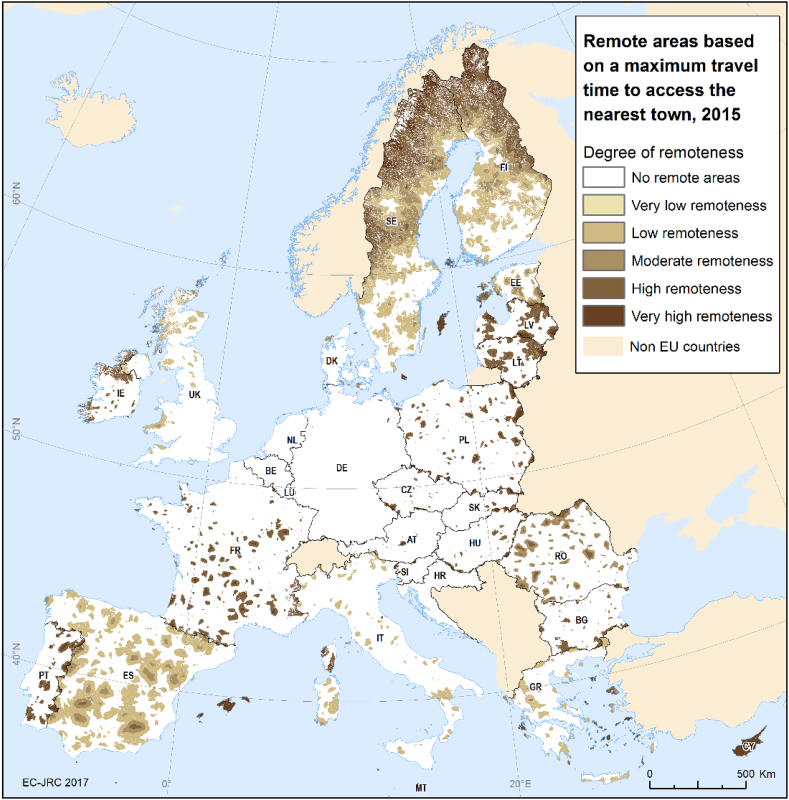
Map of remote areas computed at Member State level, adding also the UK, 2015.

Two dichotomous variables are used to flag demographic and geographic factors that increase agricultural abandonment risk. Those variables indicate places with low population density, and places that are remote (Appendix B Table B.3; Appendix C Fig. C.4 and C.5). Areas with a population density below 50 inhabitants/km^2^ are considered very low-density areas (Terres et al., 2015). Remote areas are identified as areas that are more than 60 min driving away from the closest city or town (Dijkstra and Poelman, 2008). Several studies (Corbelle-Rico and Crecente-Maseda, 2014; Corbelle-Rico et al., 2012; Gellrich and Zimmermann, 2007; Lange et al., 2013) also highlight the fact that low population density and remoteness increase abandonment risk. In low-density areas, infrastructure and public services are scarce and presumably relatively inefficient. Remote areas are characterized by limited economic opportunities and greater difficulties to reach markets; thus, agricultural activities there face higher transport cost and reduced competitiveness. In LUISA, both local population densities (a model output) and travel times (based on expected infrastructure investments) typically change throughout the simulation period, so that the demographic and geographic aspects in the compound abandonment risk map are not held fixed in the model.

#### 2.3.4 Creating a compound risk map

The last step for creating a compound agricultural abandonment risk map is the combination of the factor maps described in this section, as Table 1 shows. The spatial combination is done through weighted linear addition (WLA), with scores and weights assigned to each criterion. Particularly, the biophysical risk map is assigned the highest weight^11^ following the assumption that natural constrains set the primary pre-conditions for agricultural abandonment. The values of the final composite risk map, ranking from 0 to 100, is classified into five categories of abandonment risk following equal intervals: very low (0%–20%), low (20%–40%), moderate (40%–60%), high (60%–80%) and very high (80%–100%).

### 2.4 Validation of the European risk map and the agricultural land abandonment

**Fig. 1:**
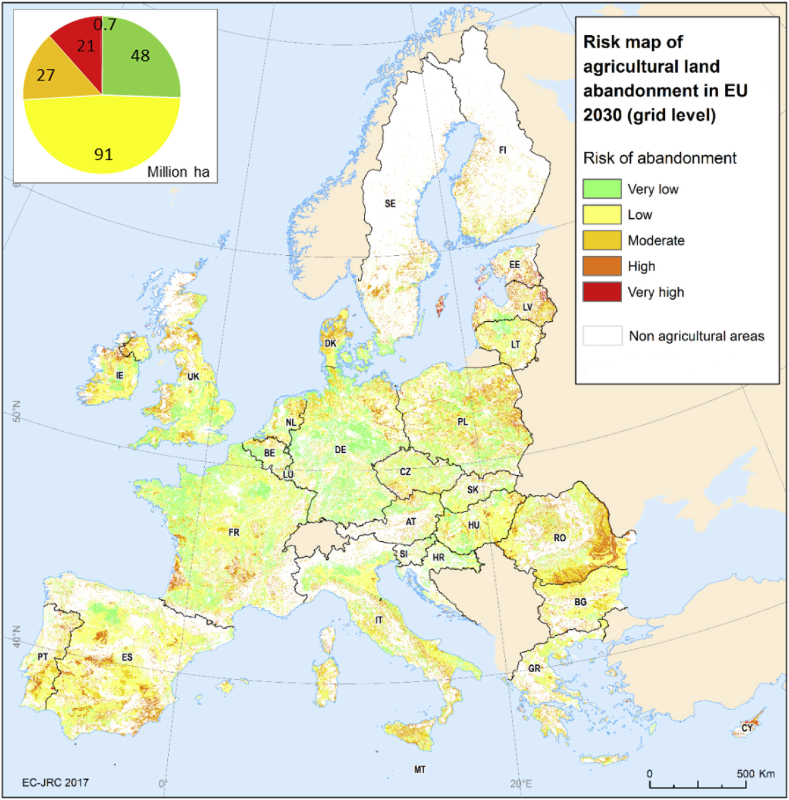
Map of the potential risk of agricultural land abandonment in 2030 at grid level (100-m resolution) in the EU and the UK. The coloured pie (top left) represents the total amount of agricultural land (Million hectares) corresponding to each aggregated risk class.

**Fig. D.1:**
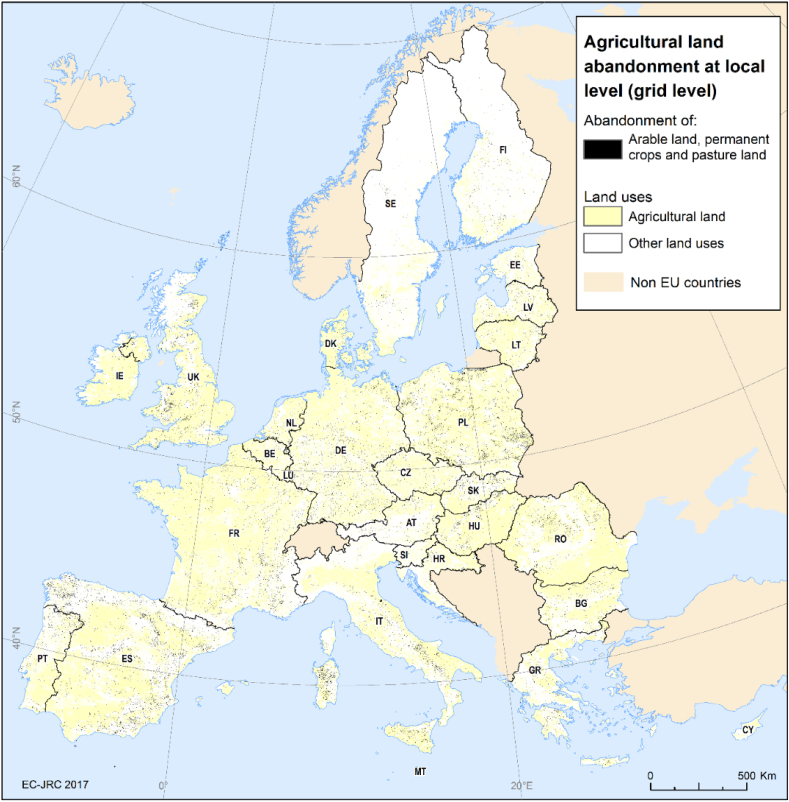
Future projections of agricultural land abandonment at grid level (100-m resolution) in the EU and the UK,2030.

A number of validation procedures were applied with the main purpose of evaluating the quality of the map in identifying either the risk of abandonment (Fig. 1) or the projected abandonment in a specific location (Appendix D, Fig. D.1). Three different strategies were applied in this validation exercise. A first approach entailed a comparison between the LUCAS^12^ (LUCAS database, 2015) and the abandonment risk map. Observed abandoned land points from LUCAS database (389 points) were overlapped with the five abandonment risk classes from the risk map. In addition, non-abandoned agricultural points from LUCAS (79,769 points) were also analysed, in order to identify the potential abandonment risk of those. Furthermore, the local extent of agricultural abandonment, as reported in Lasanta et al. (2016), was compared with aggregated municipal agricultural abandonment extents as modelled in LUISA. Lastly, to verify the assumed relevance of the factor effects of which the abandonment risk map is composed, a multivariate explanatory model is fitted to quantify the contribution of the selected factors to agricultural abandonment. To do so, all agricultural points (both abandoned and not abandoned) are selected from the LUCAS database. The risk of abandonment in that subset is subsequently explained using the point values of all biophysical and economic factors (Table 1) with which the abandonment risk map is composed. A binomial logit model (Eq (4)) is used to estimate the effects of all variables, so that

(4) $$\;P\left(abandoned\right)=\frac{1}{\text{exp}\left(\beta_{0}+\beta_{k}X_{k}+\varepsilon \right)}$$

where P refers to the probability of abandonment, β0 refers to the intercept, $\beta_{k}$ is a vector of coefficients (effect) to be estimated, $X_{k}$ refers to the independent variables and ε is the error term. Thus, the contribution of all factors towards agricultural abandonment is quantified explicitly here. All variables are defined as boolean factors where “1” (true = variables meet the criteria) represents “severe risk of agricultural abandonment” and “0” represents “no risk”. To make spatially compatible our variables in the model prediction, all variables were transformed on a pixel basis (100-m resolution) in raster layers.

## 3 Results

### 3.1 European risk map of agricultural land abandonment

In 2030, almost 183 million ha of agricultural land are projected to be under different levels of potential risk of abandonment in the EU and the UK (Fig. 1). The very large majority of that agricultural land will, nevertheless, be under very low (25%) and low (50%) risk of abandonment. About 14% of the agricultural land is estimated to be under moderate risk of land abandonment. This still leaves 11% and 0.4% (700 Kha) of the agricultural land under high and very high potential risk of abandonment, respectively.

**Fig. C.5:**
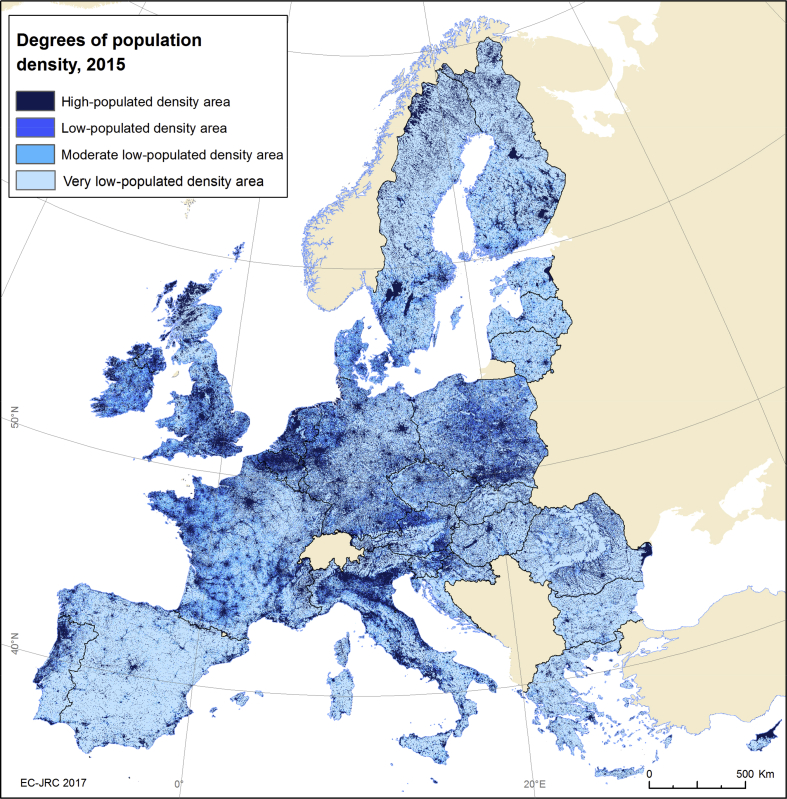
Map depicting (low) population density areas in the EU countries and the UK, 2015.

Abandonment-prone areas are dispersed across Europe, linked to variation in the presence of risk factors. Biophysical factors (Appendix C, Fig. C.1) appear to be the leading factor in large areas of Austria, Poland, Greece, Spain, Estonia and Latvia, northern parts of Sweden, Finland, Italy, Ireland, southern parts of France and Bulgaria, particularly in regions with a mountainous character (the Apennines, Pyrenees, Alps, Dolomites, Carpathians, the Central Massif in France, or the Iberian and Cantabrian mountains). Considerable abandonment risk due to climate limitations is mostly found in Mediterranean countries where soils suffer from drought (like in Greece, Italy, Spain), but also in the United Kingdom and Scandinavia, due to conditions promoting acidic and waterlogged soil conditions. Remoteness and low population density appear to be the major drivers of abandonment risk in the inner part of Spain, the middle and northern areas of Sweden, Finland and Ireland, the northern and eastern parts of Romania, and partially in Estonia, Latvia and Lithuania, Hungary and Cyprus (Appendix C, Fig. C.4 and Fig. C.5). Economic and structural farm factors (Appendix C, Fig. C.2 and Fig. C.3) are primary causes for the high agricultural abandonment risk in many regions of Spain, the north of France, Greece and Italy, the central and northern parts of Sweden and Finland, Eastern Bulgaria, as well as in Estonia, Latvia, Lithuania and Hungary.

**Fig. 2:**
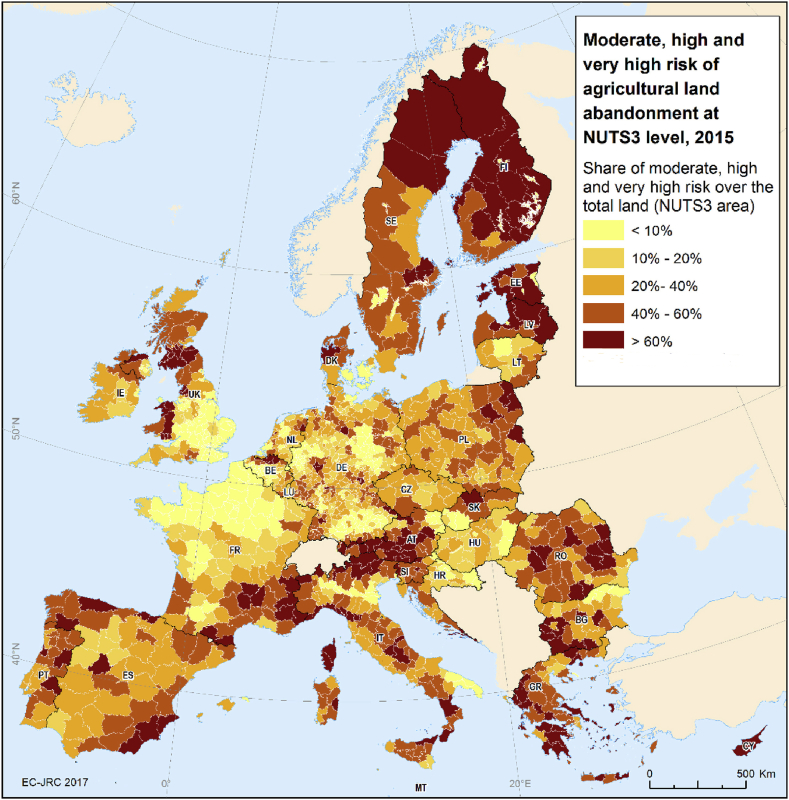
Share of moderate, high and very high risk of agricultural land abandonment as a proportion of total land in the EU and the UK.

Fig. 2 shows shares of land under moderate, high and very high risk of abandonment in proportion to regional area. Clearly, the risk of land abandonment is not limited to mountainous areas and other vulnerable regions can be identified. Several regions accounting for more than 60% of the total surface under a high risk we identified in the northern part of Portugal, Spain, Italy, Latvia, Estonia, Sweden, Finland, Austria and Bulgaria.

### 3.2 Projections of agricultural land abandonment in LUISA: from European to local scale

In the EU and the UK, agricultural land is projected to be abandoned at an average rate of 373 Kha per year, reaching roughly 5.6 Mha and accounting for approximately 3.6% of total agricultural land by 2030. Arable land is expected to be the most prone to abandonment, accounting for more than 70% of all abandonment in 2030 (4 Mha). Pastoral land (20%, 1.2 Mha) and permanent crops (7%, 400 Kha) make up smaller portions of total abandoned land. Almost a quarter (1.38 Mha) of all agricultural abandonment will most likely occur in mountainous areas^13^ where arable land would be the most affected agriculture system (974 Kha, i.e. 70% of all mountainous abandonment).

**Fig. 3:**
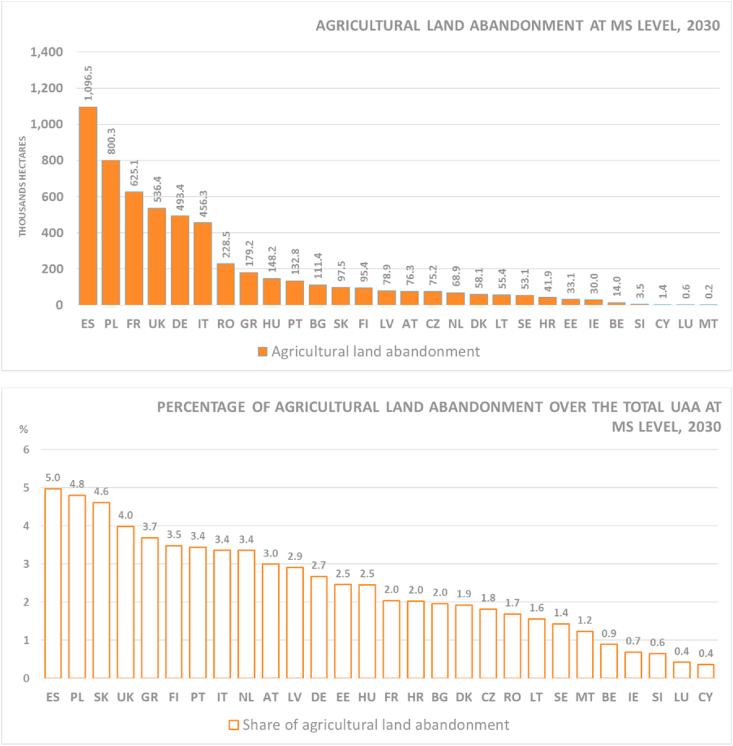
Absolute (top) and relative (bottom) agricultural land abandonment between 2015 and 2030, in EU countries and the UK.

Fig. 3 presents absolute and relative extents of agricultural land abandonment between 2015 and 2030. Spain and Poland are likely to endure the most agricultural land abandonment both in absolute and relative terms. Spain is the only studied country expected to lose more than 1 million ha, alone accounting for about 20% of all simulated losses). In terms of absolute figures, France, Germany and Italy complement Spain and Poland in the group of the largest agricultural land abandonment in the EU, altogether responsible for more than 70% of all losses. Conversely, due to their relatively smaller total agricultural land, the Netherlands, Portugal, Finland, Greece and especially Slovakia are expected to be above the 3% EU average.

**Fig. 4:**
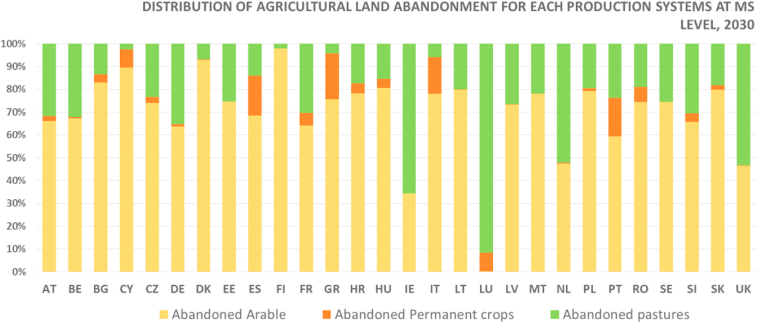
Breakdown of agricultural land abandonment (percentage) in the EU countries and the UK, 2030

Landscapes and agricultural production systems vary considerably among EU MSs, and as a consequence, so are national compositions of abandonment (Fig. 4). Abandonment of arable land is expected to be leading mode of abandonment in Bulgaria, Cyprus, Denmark, Finland, Hungary, Lithuania and Slovakia, while abandonment of pastures will be predominant in Ireland, the Netherlands and Luxembourg. Permanent crops will account for a significant share, albeit not predominant, in Southern European countries.

**Fig. 5:**
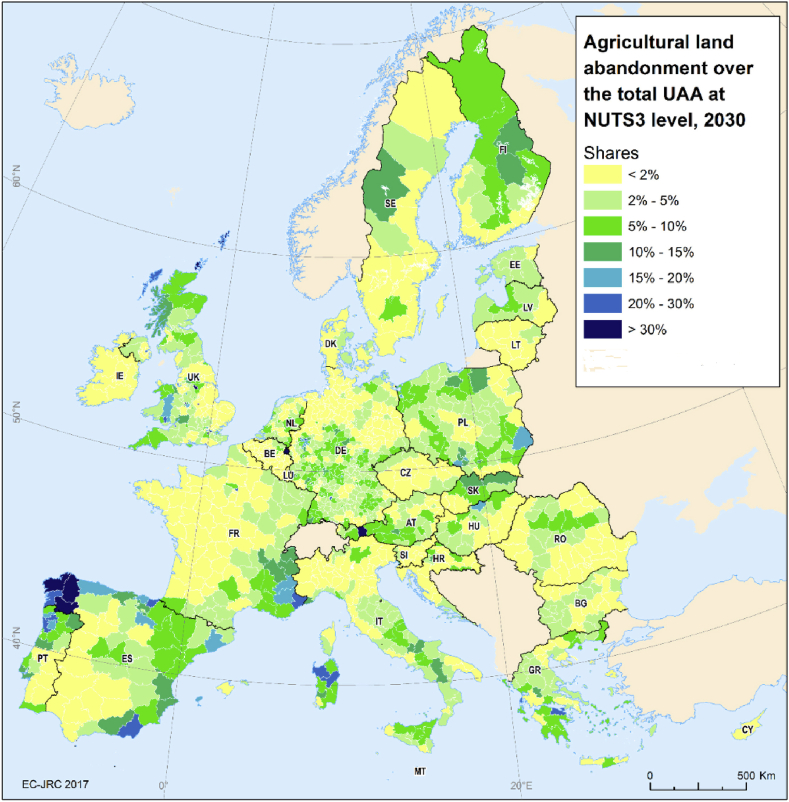
Expected shares of agricultural land abandonment with regard to the total agricultural land aggregated at NUTS3 regional level in the EU and the UK, 2030.

At the regional level, Fig. 5 presents the projections of abandoned agricultural land as share of total agricultural land aggregated at NUTS3 level in 2030. It confirms that Spain is expected to face the biggest challenges in the EU, especially in its North/Northwest. Other regions in Southern Europe are also likely to face significant land abandonment, such as Northern Portugal^14^, Southeastern France^15^, Sardinia^16^ in Italy, and Greece.^17^ In Central and Northern Europe, substantial agricultural land abandonment is projected for Western Germany, as well as in the Northern Hungary and Southeastern Poland where the largest absolute projected loss is found for the Chelmsko-zamojski region (more than 85 thousand ha). It is also worth noting that single regions in Western Austria (Innsbruck, AT332) and Southern Netherlands (Zuid Limburg, NL423) are expected to undergo a significant (more than 30%) agricultural land abandonment, though this trend is not likely to spread to the surrounding regions.

**Fig. 6:**
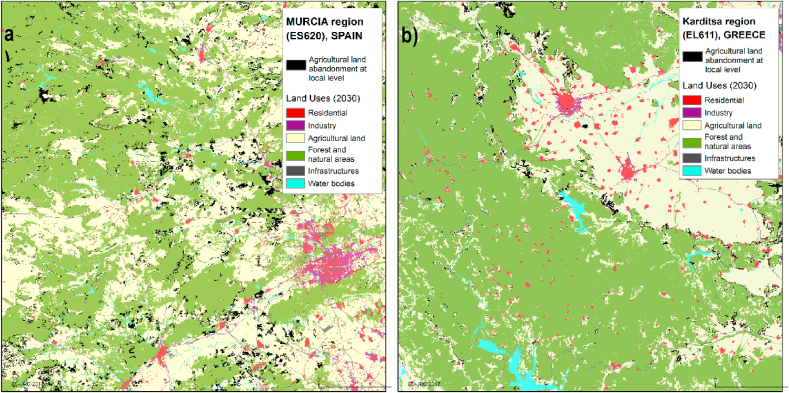
Two zooming areas where black polygons represent abandonment overlapping other land uses. a) Murcia region is Spain and d) Karditsa region in Greece.

The modelling exercise allows us to analyse agriculture land abandonment at the local scale for the whole EU territory (Fig. 6). For illustrative purposes, two zones where selected to exemplify areas affected by abandonment in Spain (Murcia) and Greece (Karditsa). Northwestern from Murcia city (Fig. 6a), a substantial amount of fruit trees, the predominant permanent crop, are expected to be abandoned. However, arable land is also abandoned, in particular close to urban centres, which is possibly related to modelled urban expansion. A combination of factors seems to drive abandonment processes in this region. Some of the agricultural land is relatively remote (more than 60 min to access the nearest town), particularly in the western part. This part of the region is also considered partially mountainous according to less-favoured area criteria. Further adding to abandonment risk, the Murcia region is also characterized by areas with high salinity concentration and low annual precipitation.

The Karditsa region (Fig. 6b), is characterized by farms with a moderate stability and viability, increasing abandonment risk. The areas of this region that are most prone to be abandoned are remote and partially or totally mountainous, and in addition, combine at least three biophysical factors (slope higher than the range 15%-30%, heavy clay texture, and low length of growing period) that increase the risk of being abandoned. This leads to substantial expected abandonment in the region, mostly affecting arable land, along with occasional patches of permanent crops (vineyards).

### 3.3 Flows of land from agricultural land abandonment to other (aggregated) land uses. Trends of agricultural land vs abandonment

**Fig. 7:**
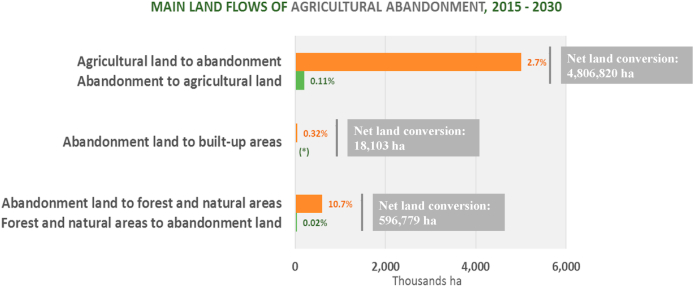
Main land-use/cover flows from agricultural land abandonment to other land-use types in the EU and the UK within 2015–2030. Net land conversions between flows are also included except for the ones that do not occur (*). The shares of the flows corresponding to “agricultural land to abandonment” and “abandonment to agricultural land” are computed in relation to the total agricultural land, whilst other land flows are computed in relation to the total abandoned agricultural land in 2030.

Analysing land-use/cover flows illustrate the main land-use trajectories that are projected to occur within the simulation period. Fig. 7 reveals that the conversion from agricultural land into abandoned land (4.8 Mha or 2.7% of total agricultural land) will dominate the inverse conversion of abandoned land for agricultural purposes (200 Kha or 0.11% of abandoned land), leaving a net conversion of about 4.8 million ha as loss of agriculture land. At 600 Kha, the conversion from abandoned land into forest and natural areas is projected to be much larger, entailing more than 10% of recuperation. The creation of new built-up areas is likely to be much less important, recovering just 18 thousand ha (about 0.3%) of abandoned agricultural land between 2015 and 2030.

**Fig. 8:**
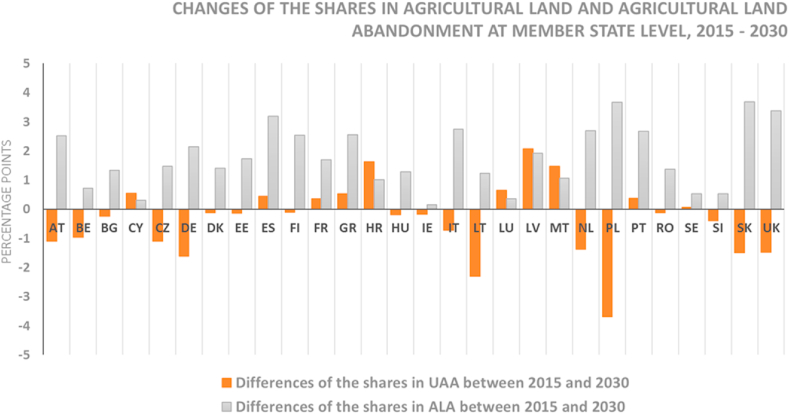
Comparison between the growth of the total agricultural land (UAA) and agricultural land abandonment at MS level, adding the UK, from 2015 to 2030.

Comparing differences in shares (agricultural land vs agricultural land abandonment) from 2015 to 2030 provides important findings at the country level (Fig. 8). Some countries show simultaneous agricultural land increase and abandonment, especially in Portugal, France, Greece, Malta, Spain, Croatia, Latvia, Cyprus and Luxembourg. This might indicate that agricultural production is being displaced to more productive areas within these countries. However, in Austria, Czech Republic, Germany, Lithuania, the Netherlands, Poland or Slovakia, there will be a net decrease of land occupied by agriculture, so that abandoned land is not offset by increases elsewhere.

### 3.4 Validation of the European risk map of agricultural land abandonment

As described in section 2.4, three different validation strategies are applied for the work at hand. First, we compare the risk map with observed abandoned agricultural land according to the LUCAS database. This comparison yields that 67.7% of the total abandoned points from LUCAS falls within moderate, high and very high categories (263 points), while the remaining 32.2% corresponds to low or very low categories (126). Taking into account that from the 79,769 observed non-abandoned agricultural points in the LUCAS database only 3.6% are in high or very high-risk areas, it seems clear that agricultural abandonment is more likely in areas considered prone to abandonment risk.

**Fig. E.1:**
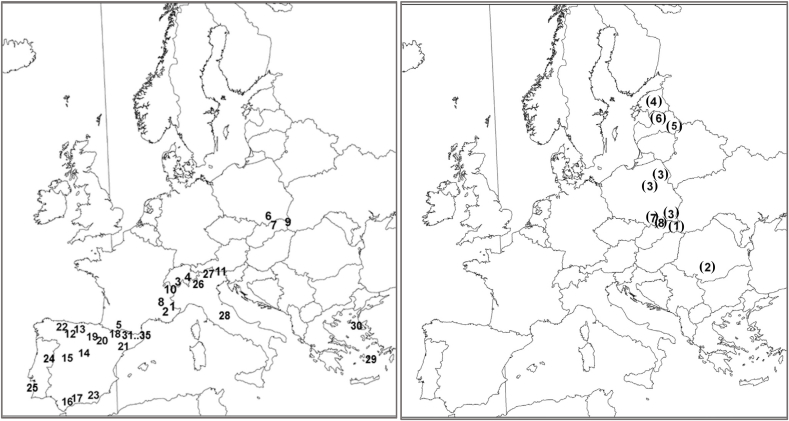
Distribution of the reported local shares of abandonment (hotspots) refers to the 35 study areas identified in Lasanta et al. (2016).

Second, we compare local abandonment hotspots compiled by Lasanta et al. (2016)^18^ (Appendix E, Fig. E.1) and areas modelled by LUISA (Appendix D, Fig. D.1), yielding considerable correspondence. A summary table of the shares of land abandonment is elaborated to easily compare measured and modelled values (Appendix E, Table E.1), though herein a detailed explanation per each country is given. In France^19^ NUTS3 regions that spatially overlap the reported mountainous areas are located in Alps (FR821-20%)^20^, Vaucluse (FR826-7%), Hautes-Alpes (FR822-18%), Hautes-Pyrenees (FR626-4%) and Isere (FR714-14%). At municipality level (LAU2), the shares of abandonment dramatically increase (up to 96% in Sigoyer, Hautes-Alpes) and the model is able to locally capture the extend and location of the reported hotspots of abandonment. Out of 34 overlapped municipalities, 16 have an abandonment share greater than 80%, similar to the abandonment reported hotspots. Spain^21^ gathers the major number of case study areas mainly due to the large distribution of mountain ranges, from the North to the South, within the Iberian Peninsula. The set of NUTS3 regions overlapping those reported abandonment hotspots are: Asturias (ES12-15%)^22^, Cantabria (ES13-14%), Guipúzcoa (ES212-20%), Madrid (ES300-9%)^23^, Ávila (ES411-8%), Salamanca (ES415-7.1%), Lleida (ES513-4.5%)^24^, Rioja (ES230-17.5%)^25^, Zaragoza (ES243-7%), Málaga (ES617-10%)^26^, Granada (ES614-12%), Almería (ES611-20.5%). 58 municipalities are analysed at local level in which abandonment shares ranging from 30% to 84%, with the highest affected areas by abandonment in Lleida (El Pont de Suert, 84%), North of Madrid (Navarredonda, San Mames and Puentes viejas, 80%), and Zaragoza (Borja and Ainzon, 80% and 81%, respectively). In the case of Poland, Carpathian mountains and their surroundings areas (Beskid Maly), as well as the regions of Mazovia, Podkarpacikie and Podlaskie are identified as abandonment hotspots areas. In particular, NUTS3 regions of Chełmsko-zamojski (PL312-17%)^27^, Rybnicki (PL227-17.5%) and Krakowski (PL214-9%) are spatially located over those areas with the greatest shares of abandonment projected by LUISA. Focusing on agricultural abandonment at local scale, 17 municipalities are assessed with shares accounting for from 32% to 66% (the latter corresponds to Lubycza Królewska). Althought Italy is one of the countries more affected by land abandonment, this fact is not reflected in the number of reported study areas^28^ These areas are mainly located in the Northeastern side of the Italian Alps (Belluno province) and Central Apennines (Riete province) limiting with Austrian Alps (Innsbruck: AT332-41% and Tiroler Unterland (AT335-8.3%). In Italy, only Sondrio (ITC44-3%) and Trento (ITH20-6.5%), in the north, and Riete (ITI42-6%) located in the central side, showed spatial coincidence between the two sources. At local level, 17 municipalities are found to have a good match in relation to the abandonment shares. Exceptions are Sondalo and Sernio (above 30% compared to 11.7%). In Riete region, municipalities affected by abandonment are in the range of the reported hotspots (by 30% local share) except in Micigliano that reaches 67% abandonment share. In Slovakia, we can assess only one point (number 7) placed in the Carpathian Mountains over the regions of Prešovský kraj (SK041-11%)^29^ and South Narodny Park Slovensky Kras (SK042-5%). At local level, LUISA reports much higher shares in all the municipalities evaluated (10) than the shares from the literature, with the highest abandonment share in Medzev (521671) at about 95%. The opposite situation occurs in Romania, where only one point (number 2) is observed in the area of Arges region (RO311-1%) and its municipalities mostly present abandonment shares lower than 10%. In Baltics countries^30^, especially Estonia and Latvia, several sites modestly reveals moderate/low abandonment shares. In Estonia, the region Laane-Eesti (EE004-3%)^31^ is the most affected by land abandonment, in line with what it is stated by the literature, however, it is difficult to find modelled abandonment shares greater than 6%. Shares in Latvia are even smaller but with some local picks in the central part of Vidzeme (LV008-3.5%) reaching 50% abandonment shares in some areas, as well as nearby Latgale region (LV007-5.2%) due to the location of the Daugava River basin and the presence of forestry and natural areas. The last two South European countries, Greece and Portugal, also have assigned few studied abandonment points (only two points each). Agricultural abandonment in Greece are observed in the Nisyros and Lesvos islands that spatially overlap the Άνδρος (EL422-10%)^32^ and Λέσβος-Λήμνος (EL411-2%) regions which means that LUISA capture lower values in these islands. In Portugal, the study areas were located in central inland near the River Côa and Alentejo whose NUTS3 regions concur with Beiras e Serra da Estrela (PT16J-3%) and Alentejo Litoral (PT181-0.6%). A set of municipalities (LAU2)^33^ were assessed yielding much lower modelled abandonment shares from LUISA than the observed areas.

Finally, a logit expression (see Eq. (4)) is used to quantify the effect of selected abandonment risk factors (Table 1) on observed agricultural abandonment. The results (Table 2) indicate a model with a high goodness of fit, indicating that the selected factors can be used to accurately predict the presence of abandonment. Most of the estimators have significant effects, with small p-values. In particular, slope, drainage, precipitation, LGP, root depth, Farm age (FA), farm investment (FI) are considered the most relevant variables, followed by farmer qualification, remoteness and population density. Most of the estimators with low p values have the expected sign. Thus, abandonment probability is greater with steeper slope, more precipitation, higher soil Ph, longer root depth, older mean famer age, poor famer qualification and in remote areas. And abandonment risk is greater with less farm investment, lower total subsidies and in areas with higher population density. The estimators for soil drainage and length growing period significantly yield signs that are contrary to our expectations. This can be explained due to the fact that both layers present a low spatial variability, for which the highly localised abandonment data is not sufficiently detailed. Moreover, better results could be obtained by the integration of irrigation maps as an economic factor, which presumably reduces the risk of abandonment locally, especially in semi-arid regions.

**Table 2:** Results of the logistic regression model.

Predictor	Estimate	Std. Error	Z value	p-value
(Intercept)	−6.7456	0.3262	−20.6780	***
Slope	1.4741	0.1271	11.6000	***
Soil drainage	−0.7463	0.2078	−3.5920	***
precipitation	0.5257	0.1265	4.1570	***
LGP	−0.6937	0.1734	−4.0000	***
SOM	−0.3703	0.3901	−0.9490	
Soil Ph	0.1602	0.2218	0.7220	
Root depth	0.8070	0.1478	5.4610	***
Salinity	−0.9137	0.4626	−1.9750	.
Texture	−0.0241	0.1205	−0.2000	
FA	0.0307	0.0037	8.3550	***
FQ	0.0084	0.0032	2.6460	**
FS	0.0017	0.0033	0.5150	
RP	0.0004	0.0004	1.0630	
R_UAA	0.0042	0.0025	1.6830	
FI	−0.2574	0.1635	−1.5740	
LFI	−0.0008	0.0002	−3.7860	***
TS	−0.0016	0.0004	−4.5160	***
Remoteness	0.6759	0.2506	2.6970	**
Population density	−0.1358	0.1278	−1.0630	**

Significance is indicated with ‘***’ 0.001; ‘**’ 0.01; ‘*’ 0.05; ‘.’ 0.1; ‘ ’ 1.

Note: LGP (Length of growing period), SOM (soil organic matter), FA (Famers age > 65 years old), FQ (Farmer qualification), FS (Farm size), RP (Rental price), R_UAA (Rented UAA), FI (Farm income), LFI (Level of farm investment) and TS (Total subsidies).

## 4 Discussion

Even though agricultural land abandonment is a sizeable process in Europe, a wide overview and future outlook of abandonment are missing for the continent. Previous contributions covered case studies and partial aspects of the process (García-Ruiz and Lana-Renault, 2011; Lasanta et al., 2016), which due to the considerable spatial heterogeneity of economic, biophysical and climatic conditions cannot easily be generalized for a greater geography. Determining the extent and location of agricultural abandonment requires not only agreed upon definitions, driving forces and potential impacts but also precise (observed) data, and knowledge about the transformation and subsequent dynamics of the natural revegetation (Keenleyside and Tucker, 2010; Verburg and Overmars, 2009). Clearly, the lack of an existing continuous and harmonized database/inventory of land abandonment mars deeper, more accurate analysis (Estel et al., 2015; Levers et al. 2018)^34^.

The way to model agricultural abandonment by the LUISA model differs substantially from previous abandonment modelling exercises in terms of assumptions, spatial and thematic resolution, and reference land-use data. To estimate the extent and locations of future abandonment, LUISA attempts to endogenously model agricultural land abandonment as a choice of disinvestment, using a map of induced abandonment risk to capture the most likely locations of abandonment while taking into account the spatial heterogeneity of Europe's farming conditions. The most recent available data and information were used to compose that risk map. However, a set of uncertainties are intrinsically part of this type of modelling, coming into play through choices such as thematic, spatial and temporal resolution, data availability, geographical coverage, assumptions and aggregation methods (Estel et al., 2015; Price et al., 2015). For instance, when examining factors (Table 1) by which the risk map is composed, it is evident that both the lack of higher resolution for some spatial layers (for instance, length growing period or drainage) and the low accuracy and spatial variability of some factors (salinity and sodicity) reduces the quality of the final results, as is evident in the validation exercises. Another example is posed by agroeconomic variables, which are available as regional averages. Clearly, land abandonment depends on farm-specific characteristics, and therefore regional statistics offer a limited approximation for determining the precise location of farms at risk.

An extensive exercise has been executed to validate the inclusion of the agricultural abandonment model. We find that the introduced agricultural risk map coincides with abandonment observed in the LUCAS database. Modelled abandonment coincides with the abandonment shares reported by Lasanta et al. (2016), although discrepancies can be found especially in Spain, Portugal, Italy, Poland, Slovakia and Latvia. Those discrepancies can be explained by to the fact that the abandonment hotspots reported in Lasanta et al. were measured very locally, more than two decades ago. A logit model has been used to verify whether the factors selected for the potential risk map contribute to a higher likeliness of observing abandoned agricultural areas according to the LUCAS definition. This exercise corroborates that slope, low precipitation, poor drainage, population density, travel times and distance to farms increase likeliness of abandonment (Alonso-Sarría et al., 2016; Corbelle-Rico and Crecente-Maseda, 2014). Regional farmer qualifications, shares of older farmers, farm investments and subsidies are also found to have the expected structural effect on abandonment likeliness, confirming many previous results (Keenleyside and Tucker, 2010; Prishchepov et al., 2013; Terres et al., 2015; Lasanta et al., 2016; Levers, et al., 2018). Other variables did not yield significant effects or the expected signs, which may be due to the limited amount of initial observations. Possibly, agricultural areas where the condition at hand exists are already abandoned or local strategies were applied to overcome the difficulties caused by the condition that the variable describes. Agroeconomic regional variables such as farm size, rental price, farm income and share of rented land did not yield significant results, no doubt because the local variation of those variables is substantial within a region. These variables are nevertheless included in the potential risk map as they do assist in pinpointing in which regions abandonment is more likely to occur. The variables indicating poor soil drainage and high levels of soil organic matter (SOM) yielded counterintuitive effects. The results from SOM entail a paradox, as fertile soil with high organic matter is in fact less prone to be abandoned. Agricultural areas with poor drainage may be compensated by irrigation systems, thus raising sunk costs in the farm operation and making abandonment likeliness lower. Unfortunately, the data necessary to verify this more thoroughly is unavailable or insufficient.

Despite the many differences between prior modelling approaches, LUISA's abandonment results corroborate many of the findings of previous works. The total abandonment share expected here is very similar to the 3.7% from the most moderate scenario reported by Van der Zanden et al. (2017). However, estimates of the amount of abandonment differ substantially. Recently, Estel et al. (2015) and Levers et al. (2018) mapped active cropland, fallow land and farmland abandonment, with abandoned values ranging between 0.2% and 1.4% of the total farmland. Assuming high global competitiveness and lacking public support for farming, Keenleyside and Tucker (2010) expect much more abandonment at a rate of 7%. In terms of pinpointing the locations of abandonment, again LUISA corroborates many previous results. Similar to Verburg and Overmars (2009) and Renwick et al. (2013), substantial abandonment is expected here in mountainous areas. However, LUISA also captures hotspots outside mountainous areas, particularly in the northwestern Spain (Galicia), Corsica, northwestern and central part of Portugal^35^, Baltic's countries^36^ including new detected abandoned areas in Lithuania^37^, northwest of France, northeaster and western part of Poland^38^, north of the national park Nizke Tatry (Slovakia)^39^ and in the western side of the Carpathians (immediately in the North of Arges region)^40^. Other sources are not definite on where abandonment would happen outside mountainous regions. The LUISA results are in line with locations reported by Lasanta et al. (2016)^41^ and occur especially in Poland, Romania, Baltics countries, the southeast of Spain, and the south of Portugal. Estel et al. (2015) and Levers et al. (2018) expect abandonment outside mountainous areas to occur in very different places; casting some doubt on the LUISA outcomes. Specifically for the Iberian Peninsula, their predictions are not completely coincident with regional/local studies carried out by many authors (Alonso-Sarría et al., 2016; Lasanta et al., 2016; Arnáez et al., 2011; Corbelle-Rico et al., 2012; Pinto Correia, 1993; Nunes et al., 2011); however, a more comprehensive comparison of modelling approaches and results is called for here. Lastly, due to its thematic detail and interactions between land uses, LUISA projects dynamics between land abandonment and other land uses (e.g. residential, industrial areas) that are expected to occur in particular around main capital cities such as in Paris, Madrid, Berlin or Warsaw. There, in the simulations, agricultural land becomes abandoned, possibly as a precursor for urban expansion or urban sprawl. These land conversions deserve further studies since they are likely related to the concept of “land reservoir” (Van der Zanden et al., 2017; Grădinaru et al., 2015; Paul and Tonts, 2005; Price et al., 2015).

When an abandonment process occurs, it affects not only the abandoned area itself but also its local population and the whole society in terms of production of goods (e.g., foods, feed, fibre and biomass production) as well as other services provided by the multifunctionality of the agricultural land (Elbersen et al., 2014). One of the most important function of agriculture is to feed the EU population and, likely, the food security can be one of the major challenges for the future of the EU, especially for the rural economy (Terres et al., 2015). For many regions in Europe, the agricultural sector still plays a significant economic role (ECORYS, 2010) and its eventual decline due to massive abandonment, among other factors, might cause a loss of jobs in the agricultural and related sectors, out-migration of young people and a decline in the management of agroecosystems (Lasanta et al., 2005). The decrease in agricultural land influences agricultural outputs and management practices. Changes in management practices such as agricultural intensification and specialization lead to high productivity in more fertile areas, while causing marginalisation and abandonment in others (Baumann et al., 2011).

## 5 Conclusion

Agricultural land abandonment is the largest land-use change process in Europe and it is expected to continue during the next decades. Land abandonment has been analysed in European mountainous and remote areas since the earlier decades of the 20th century, but less effort has focused on other vulnerable areas. This study, therefore, presents a comprehensive European, spatially explicit exercise to model agricultural land abandonment. This was done within the LUISA Territorial platform from the period 2015–2030 at a high spatial resolution for all EU countries and the UK. Abandonment is considered a (dis)investment decision, and the location of abandonment is defined by an abandonment risk map deduced from previous findings in the literature. That risk map was composed by combining a set of factors that presumably drive agricultural abandonment, highlighting the importance of biophysical conditions, agricultural socio-economics, farm structure, demographics and geography.

By 2030, results reveals that the total abandonment is projected to reach more than 3% (5.6 million ha) of the total agricultural land while, at the same time, the decrease of agricultural land over the same period of time is an evident fact in most EU countries and the UK. Spain and Poland are likely to account for one third of the EU total land abandonment, whereas France, Germany and Italy complement the leading group (altogether responsible for more than 70% of the total abandonment). This abandonment share is not equally spread across EU countries, ranging from less than 2% to more than 50% at the regional level. Areas that are hot-spots of undesirable abandonment might be particularly aimed at by policymakers in order to prevent or minimize present and future negative consequences, and our results can be a valuable spatial and quantitative source of information to this end.

Modelling dynamic indicators require a set of geospatial and statistical data whose availability, accessibility and resolution are often limited, potentially affecting the reliability of the data produced. Multiple strategies were followed in order to validate the implemented approach. We need to emphasize the challenge of direct comparison with other sources because for instance temporal coverage, data, assumptions, abandonment definitions and spatial location vary among all those studies. Despite differences in location and extent, we stress the considerable spatial overlapping between LUISA and other datasets and model results. Definitively, LUISA projections of abandonment, however, seems to be conservative but in line with European average figures when compared to case studies based on policy scenarios and modelling.

## Disclaimer

The views expressed are purely those of the author and may not in any circumstances be regarded as stating an official position of the European Commission.

## Declaration of competing interest

The authors declare that they have no known competing financial interests or personal relationships that could have appeared to influence the work reported in this paper.

## Figures and Tables

**Fig. A.1:**
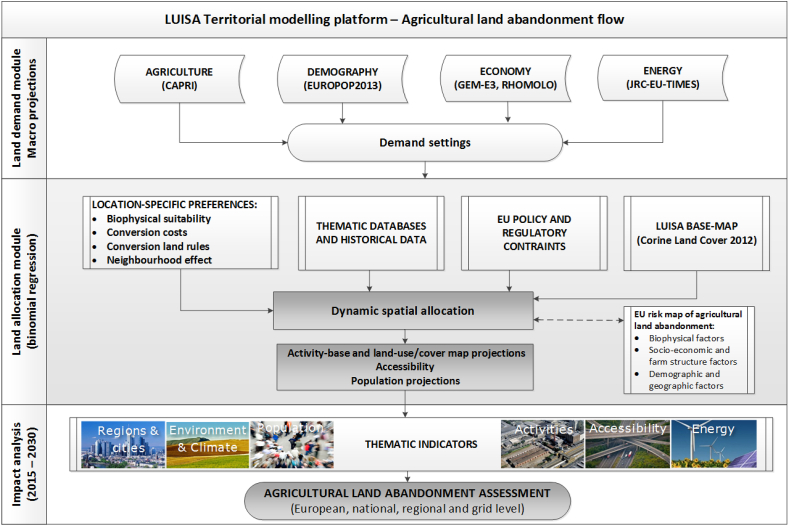
Adapted and simplified LUISA scheme to illustrate the workflow and main components for modelling, specifically, agricultural land abandonment.

**Fig. A.2:**
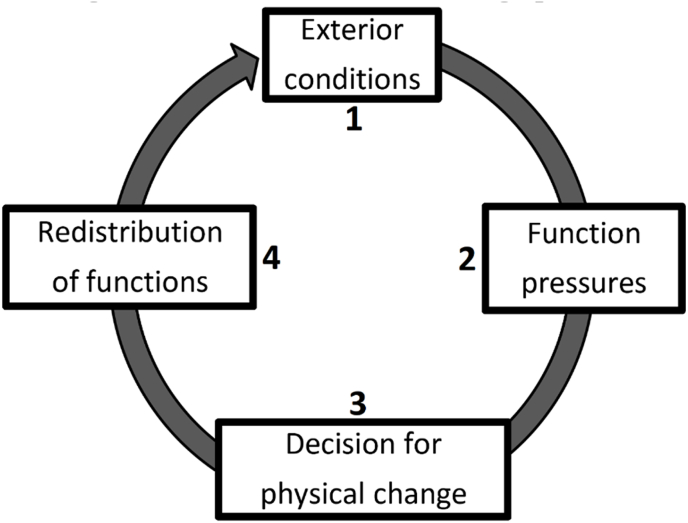
LUISA modelling framework for simulation of land-use change following pressures for the provision of land-based functions (adapted from Jacobs-Crisioni et al., 2017).

**Table B.1:** Selected biophysical criteria for identifying severe natural conditions for generic agricultural activities, their description and main reference

Biophysical land suitability factor	Description	Reference
Length of growing period	The number of days when the average daily temperature is above a certain temperature threshold. LGPt5 is selected, establishing 5 °C as threshold. Spatial resolution: 8 km (grades arc).	IIASA/FAO (2013)^a^
Organic matter	Topsoil (0–30 cm) organic matter content. Spatial and temporal resolution: 1 km; processed in 2012.	Hiederer (2016) based on ESDB and HWSD (IIASA/FAO/ISRIC/ISSCAS/JRC, 2012)
Soil drainage	It refers to the maintenance of the gaseous phase in soil pores by removal of water. Imperfect, poor and very poorly drained soils are considered not favourable for crop growth. Spatial resolution: 1 km.	SINFO project^b^ (Eurostat - European Commission, 2013) which is based on ESDB^c^
Precipitation	The total mean annual precipitation is calculated as the sum of the mean monthly precipitation. The mean annual precipitation (in mm) was divided into seven classes with 200 mm intervals, from 0 to >1000 mm. Spatial resolution: 1 km; processed in 2012.	EFSA^d^ (Eurostat - European Commission, 2013); Hiederer, R. (2016)
Soil pH	Spatial layer of topsoil pH which represent the pH given for the dominant soil. Soil pH exceeding 9 or below 4 (extreme values) is considered not favourable for crop growth. Spatial resolution: 1 km.	EFSA (Eurostat - European Commission, 2013) based on HWSD^e^ (IIASA/FAO/ISRIC/ISSCAS/JRC, 2012).
Root depth	To ensure maximum root development due to the presence of specifics horizon that cannot be penetrated by the roots. This spatial layer is divided into eight classes from <10 to >120 cm. Spatial resolution: 1 km.	SINFO project^f^ (Eurostat - European Commission, 2013) which is based on ESDB^g^
Soil texture	Five classes were defined: coarse, medium, medium fine, fine and very fine. Soil texture with less than 18% clay, more than 65% sand, or which have stones, boulders or rock at the surface are considered not favourable for crop growth. Spatial resolution: 1 km.	SINFO project (Eurostat - European Commission, 2013) which is based on ESDB
Slope	Derived from the elevation was divided into six classes. Flat areas or with a slope <8% are the most appropriated for crop growth. Slopes in excess of 16% will provide difficulty for harvesting machinery. Spatial and temporal resolution:100 m, 2013.	Shuttle Radar Topographic Mission (NASA 2013)
Salinity	Medium or high salinity concentration areas are proposed as unfavourable agricultural conditions producing significant losses of production and serious damage to the crop. Spatial resolution: 1 km.	SINFO project (Eurostat - European Commission, 2013) which is based on ESDB.
Sodicity	Soil sodicity is a land characteristic for which the proportion of absorbed sodium in the soil clay fraction is too high for plants to perform or survive. Spatial resolution: 1 km.	SINFO project (Eurostat - European Commission, 2013) which is based on ESDB.

a IIASA (International Institute for Applied Systems Analysis) and FAO (Food and Agricultural Organization of the United Nations).

b SINFO project (Soil Information System for the MARS Crop Yield Forecasting System).

c ESDB (European Soil Data base).

d EFSA (European Food Safety Authority, Spatial Data Version 1.1).

e HWSD (Harmonized World Soil Database).

f SINFO project (Soil Information System for the MARS Crop Yield Forecasting System).

g ESDB (European Soil Data base).

**Table B.2:** Main farm structure and agricultural viability factors that drives agricultural land abandonment, their description and main reference

Economic viability of agricultural production	Description	Reference
Age of farmers	It is computed as a share by taking into account the number of farmers >65 year old over the total number of farmers. It is assumed that abandonment is more likely to occur when the farmer is close to the retirement age. Factor interpretation: The higher the share of farmers older than 65 years old, the higher the abandonment risk. Spatial resolution: NUTS3 level	EUROSTAT – FSS (Eurostat - European Commission, 2017) Data: Holders above 65_ef_r_farm2007.xls
Farmer qualification	Three different levels of training of farm managers are specified: with only practical experience, basic and full agricultural training. It is computed as a share of farmers with practical experience with regard to the total number of trained farmers. Farmer with high qualification invest more in human capital, knowledge, etc., thus preventing farmland abandonment. Factor interpretation: The higher the share of farmer no qualified (only practical experience), the higher the abandonment risk. Spatial resolution: NUTS0 level	EUROSTAT (Eurostat - European Commission, 2017). Data: Total_ef_mptrainman.xls Practicalexperience_ef_mptrainman.xls
Farm size	Share of farms (UAA) under 50% of the average size region (NUTS3). The rationale behind is that larger farms can share agricultural resources (machinery, inputs, buildings, etc.) and, thus, reducing production costs. In this way, large farms compared to small (fragmented) farms are usually more competitive and viable from an economic point of view. Factor interpretation: The higher the share, the higher the abandonment risk. Spatial resolution: NUTS3 level	EUROSTAT – FSS (Eurostat - European Commission, 2017) Data: Size and type_ef_r_farm-3.xls
Rent paid	Rent paid (SE375)^a^ for farmland and buildings and rental charges^b^. Rent paid is used as a proxy of the strength or weakness of the land market. It is assuming that high rental prices leads to high demand for agricultural land and therefore, a low risk of abandonment. Units: Euro. Factor interpretation: The lower the rental price, the higher the abandonment risk. Spatial resolution: *NUTS –samples*	FADN dataset and DG AGRI RICA (especial request)
Rented UAA	Utilized agricultural areas rented (SE025) by the holder under a tenancy agreement for a period of at least one year (remuneration in cash or in kind). It is expressed in hectares (10,000 m^2^)^5^. It is computed as a share of the rented UAA over the total UAA. The average is calculated for the years 2005–2010 for each holding in the database. Units: ha Factor interpretation: The lower the rented UAA, the higher the abandonment risk. Spatial resolution: *NUTS –samples*	FADN dataset and DG AGRI RICA (especial request)
Farm income	Farm Net Value Added (SE425) expressed per agricultural work unit. Takes into account any differences in the labour force to be remunerated per holding^5^. This variable is used as a proxy of economic performance compared to the gross domestic product (GDP) per capita from the period 2005–2010. National GDP is a proxy of national income. Units: Euro. Factor interpretation: The lower the income, the higher the abandonment risk. Spatial resolution of GDP:NUTS0 Spatial resolution of Farm Net Value Added: *NUTS -samples*	FADN dataset and DG AGRI RICA (especial request) EUROSTAT (Eurostat - European Commission, 2017). Data: nama_gdp_c.xls (Euros/capita at market price)
Farm investment	Net investment (SE521) is defined as Gross investment – Depreciation^5^. This variable is normalized by the size of the farm (UAA)^c^ at sample level. This can be interpreted as a proxy of improving (new machinery, new technics) and continuing farm activities, hence reducing the risk of abandonment. Units: Euro. Factor interpretation: The lower the investment, the higher the abandonment risk. Spatial resolution: *NUTS -samples*	FADN dataset and DG AGRI RICA (especial request)
Farm scheme (subsidies)	Subsidies on current operations linked to production (not investments). Interest subsidies and payments for cessation of farming activities are therefore not included^5^. The indicator is computed by using the variable “Farm subsidies” (SE605) normalize by the UAA sample area. Units: Euro. Factor interpretation: The lower the subsidies, the higher the abandonment risk. Spatial resolution: *NUTS -samples*	FADN dataset and DG AGRI RICA (especial request)

a Codes assigned by FADN for the selected variables.

b Defined by FADN (European Commission, 2000).

c UAA not include areas used for mushrooms, land rented for less than one year, woodland and the other farm areas (roads, ponds, non-farmed areas, etc.). It is made up of land in owner occupation, rented land, land in sharecropping (remuneration linked to output from land made available). It includes agricultural land temporarily not under cultivation for agricultural reasons or as a result of being withdrawn from production as part of agricultural policy measures.

**Table B.3:** Main demographic risk factors favouring farmland abandonment: low population density and remote areas

Demographic and regional context	Description	Reference
Low population density areas	Population density below 50 inhabitants/km2 is considered low populated areas in our study, being much lower than the threshold used in other methods (150 inhabitants/km2). Several dynamic test were done to set up this cut-off value in order to better capture rural areas with very low population density. The modelling mechanism counts for each cell the allocated residents within a surrounding kernel with an area of (approximately) 1 km^2^; then, It is possible to identify the cells with less than 50 inhabitants inside the surrounding kernel.	LUISA population density map based on EUROPOP2013 (NUTS3 level) Terres et al. (2015)
Remoteness	Remote areas are represented as a dynamic map of travelling time to nearest town. Town access is defined as dynamic map of travelling time to the nearest town. Couple with access to the nearest town, this indicator also takes into account changes in travelling times between time steps. Thus, remote areas are identify as those that are further than 60 min away from towns.	Dijkstra and Poelman (2008), Terres et al. (2015)

**Table E.1:** Simplified version of the table from Lasanta et al. (2016) (first five columns) combined with the LUISA results (regional and local level) in 2030 (last two columns). Additional hotspots abandonment areas captured by LUISA are also reported (in bold).

Code	Country	Locations	Share of farmland abandonment	Reference	Regional (NUTS3) share of abandonment from LUISA 2030	Local (LAU2) share of abandonment from LUISA 2030
1	France	Alps (Briançonnais)	79%	in Lasanta et al., 2016	Alps (FR821) - 20%	52%–61%
2		Pre-alps (Provence)	97%	in Lasanta et al., 2016	Vaucluse (FR826) - 7% Hautes-alpes (FR822) - 18%	53%–94% 50%–96%
5		Central Pyrenees	84.1%	in Lasanta et al., 2016	Hautes-pyrenees (FR626) - 4%	64%–91%
8		Pre-alps	20%	in Lasanta et al., 2016	Isere (FR714) - 12%	60%–88%
32 (3)		Mazovia, podkarpacikie and podlaskie	17.6%	Keenleyside and Tucker (2010)	Chełmsko-zamojski (PL312) - 17%	43%–66%
36 (7)	Poland	Beskid Maly	33%		Rybnicki (PL227) - 17.5%	34%–51%
6 (1)	Slovakia, Poland and Ukraine	Carpathian mountains Carpathian mountains and surroundings	20% 20.7%, 13.9% and 13.3%	in Lasanta et al., 2016	Krakowski (PL214) - 9%	31%–41%
**9 (8)**				**LUISA platform**	**Olsztyński (PL622) - 11%** **Szczecinecko (PL427) - 9.5%**	**38% - 50%** **37% - 43%**
7	Slovakia	Carpathian mountains	20%	in Lasanta et al., 2016	Prešovský kraj (SK041) – 11% South Narodny park Slovensky Kras (SK042) −5%	61%–90% 61%–91%
				**LUISA platform**	**National Park Nizke Tatry (North)** **Žilinský kraj. (SK031) – 10%**	**25% -95%**
10	Italy, Austria, France, Germany, Switzerland	Alps	20%–70%	in Lasanta et al., 2016	Haute-Savoie (FR718) – 10.4%	26%–95%
11	Austria, Italy	Eastern-Central Alps	40%	in Lasanta et al., 2016	Innsbruck (AT332) – 41% and Tiroler Unterland (AT335) – 8.5%	31%–61%
12	Spain	Cantabrian mountain (South)	40%	in Lasanta et al., 2016	Asturias (ES120) – 15.2% Cantabria (ES130) – 14% País Vasco (Montes Vascos) - 20%	43%–53% 38%–48% 32%–63%
13	Spain	Cantabrian mountain (Nord)	50%	in Lasanta et al., 2016		
22	Spain	Cantabrian mountain	80%	in Lasanta et al., 2016		
14	Spain	Central System	89%	in Lasanta et al., 2016	Sierra de Ayllon and North of Madrid (ES300) - 8.7%	42%–80%
15	Spain	Central System	75%	in Lasanta et al., 2016	Ávila (ES411) and Salamanca (ES415) Salamanca - 7.1%	31%–78%
16	Spain	Sistema Bético	36%	in Lasanta et al., 2016	Malaga (ES617) - 3.8%	10%
18	Spain	Central Pyrenees	71%	in Lasanta et al., 2016	Lleida (ES513) - 4.5% Teruel (ES242) - 9.3% Tarragona (ES514) - 8.7%	53%–85%
21	Spain	Catalan Prelitoral Mountain	40%	in Lasanta et al., 2016		31%–65%
19,20	Spain	Iberian Range and Iberian Range	99% 42%	Arnáez et al. (2011) in Lasanta et al., 2016	Rioja (ES230) - 17.5% Zaragoza (ES243) - 7%	33%–57% 31%–81%
23	Spain	Sistema Bético (Alpujarras)	70%	in Lasanta et al., 2016	Sierra Nevada (ES614) – 12% Almeria (ES611) - 20.5%	53%–74% 54%–64%
**ES**	**Spain**	**Macizo galaico y Montes de Leon**	**-**	**LUISA platform**	**North of Portugal** **Lugo (ES112) 44%** **Ourense (ES113) - 41%** **Pontevedra (114) - 26%** **A coruña (ES111) - 30%**	
**ES**	**Spain**	**Catalan Prelitoral Mountain**	**-**	**LUISA platform**	**Navarra (ES220) – 6%,** **Guipuzcoa (ES212) – 29% and**	
**ES**	**Spain**	**Iberian Range and Iberian Range**	**-**	**LUISA platform**	**Teruel (ES242) - 9.3% and Castellon (ES522) - 9% in the South of Iberian Range**	
24	Portugal	Central inland	80%	Nunes et al. (2011)	Beiras e Serra Estrela (PT16J) - 3%	20%–60%
25	Portugal	Alentejo	40%	Pinto Correia (1993)	Alto Alentejo (PT186) - 1%	5%–6%
**PT**	**Portugal**	**Lisbon**	**-**	**LUISA platform**	**Lisboa (PT170) – 10%** **Parque Nacional de Peneda, C** **Cávado (PT112) – 20% and Ave (PT119) – 21%** **Oporto (PT11A) – 21%**	**56% - 85%** **50% - 65%** 43%–49%
26	Italy	Alps	11.7%	in Lasanta et al., 2016	Sondrio (ITC44) - 3%	8%–37%
27	Italy	Eastern Alps	40%	in Lasanta et al., 2016	Trento (ITH20) - 10%	29%–48%
28	Italy	Apennines	26.6%	in Lasanta et al., 2016	Rieti (ITI42) - 6%	25%–67%
**IT**	**Italy**	**Torino** **Sicilia**	**-**	**LUISA platform**	**Teramo (ITF12) - 5.7%** **Napoli (ITF33) - 5%** **Avellino (ITF34) - 9%** **Torino (ITC11) – 1.3%** **Messina (ITG13) - 6%** **Catania (ITG17) - 5%** **Reggio di Calabria (ITF65) – 3.4%**	31%–66% 31%–68% 33%–83%
29	Greece	Nisyros Island	76.4%	in Lasanta et al., 2016	Κάλυμνος, Κάρπαθος, Κως, Ρόδος (EL421) - 3.4%	52%
30 **EL**	Greece **Greece**	Lesvos Island **North and central Greece, Central Peloponeso**	20% –	in Lasanta et al., 2016 **LUISA platform**	Λέσβος, Λήμνος (EL411) - 2% **Κορινθία (EL652) - 20%** **Αργολίδα, Αρκαδία (EL651) - 9%** **Καρδίτσα, Τρίκαλα (EL611) - 5%**	38%–45% 37%–71% 28%–41.6% 42%–90%
31 (2)	Romania	Arges county	21%	in Lasanta et al., 2016	Arges (RO311) - 1%	
	**Romania**	**Central Romania**	–	**LUISA platform**		
33 (4)	Estonia	Several sites	10.1% and 30%	in Lasanta et al., 2016	Lääne-Eesti (EE004) - 3% (western and Southwestern part)	28%–40%
34 (5)	Latvia	Latgale and other regions	10.3%, 21.1% and 50%	in Lasanta et al., 2016	Pieriga (LV007) - 5.2% (river)	6%–11%
35 (6)	Latvia	Vidzeme	25%–35%	in Lasanta et al., 2016	Vidzeme (LV008) - 3.5%	15%
**LV**	**Latvia**		**-**	**LUISA platform**	**Zemgale (LV009) – 2.6% (South)** **Kurzeme (LV003) – 2.7% (West)**	**12%** **12% - 16%**

*">(*) Codes represent the studied abandoned areas in Fig. E.1.

**">(**) Share of abandonment represents the minimum and maximum local value within the corresponding NUTS3 region overlapping the studied abandoned point (hotspot areas from Lasanta et al., 2016).

## Appendix A Description of LUISA

LUISA coherently links specialised macro-economic, demographic and geospatial models with other thematic spatial/statics databases in order to simulate the local allocation of land functions (i.e. housing, transport, food production or ecosystem services). Land claims are provided by those models that govern the starting stage of the simulation, primarily, based on DG ECFIN^42^ projections. In particular, demographic projections rely on EUROPOP 2013^43^ for the period 2015–2060 at national level. CAPRI model (Britz and Witzke, 2012) provides future agricultural land demands aiming at modelling changes in CAP (Common Agricultural Policy) policies. Economic projections are derived from the GEM-E3 and the RHOMOLO model, while energy projections depend on the JRC-EU-TIMES model.

The reference spatial map, named the LUISA base map 2012^44^, is an enriched version of Corine Land Cover (2012) but with a significantly higher spatial and thematic resolution mainly owing to the integration of relevant land use/cover information from multiple compatible geodata sources^45^. Model run, therefore, provides outcomes at local scale (100-m resolution) with a temporal resolution of 5 years for all EU countries and the UK throughout a simulation period (currently, up to 2030). Sectoral claims are allocated over the land according to their specific location and recent land-use class, land suitability, restrictions of spatial policies, transition rules, neighbourhood effects, etc. More information about LUISA application, key components, modular structure and methods can be found in Jacobs-Crisioni et al. (2017), Baranzelli et al. (2016) and Lavalle (2017). Figure A.1 shows a simplified scheme of the LUISA platform in order to reflect top-down dynamics in modelling agricultural land abandonment while competing with other land uses.

The allocation mechanism is located at the core of the LUISA's modelling platform as a system of equations that repeats itself every five years until the end of the simulation period is reached, typically in 2050, following the next steps as shows Figure A.2:

1. describe the geographical context of every modelled region and grid cell, by mapping a large breadth of exogenous and endogenous external factors;
2. describe function pressures, in particular what is considered as the ‘population pressure’, or the intrinsic attractiveness of a grid cell for human settlement;
3. reallocate the physical manifestation of land uses, based on a special case of the doubly constrained land-use optimization mechanisms described in Hilferink and Rietveld (1999), and land-use specific local utility estimates computed as described in Koomen et al. (2015); and lastly,
4. redistribute land-based functions, in particular population based on environmental characteristics and reallocated land-use maps.

## Footnotes

1 See, for instance, definition of abandoned agricultural land in Hart et al. (2013) referred to actual abandonment, semi-abandonment or hidden abandonment and transitional abandonment, Pointereau et al. (2008) or Corbelle-Rico and Crecente-Maseda (2008). In Terres et al. (2015) farmland abandonment was defined as a cessassion of land management, which leads to undesirable changes in biodiversity and ecosystems services.

2 Spain, Italy, France, Greece, Portugal. Although it is possible to find studies on land abandonment in most of the EU countries (Germany, The Netherlands, Poland, etc.), which refer to local or regional scales.

3 Links to LUISA web platform (European Commission- Joint Research Centre):Urban data Platform: http://urban.jrc.ec.europa.eu/Territorial Dashboard: http://urban.jrc.ec.europa.eu/t-board/index.htmlSTRAT-Board: http://urban.jrc.ec.europa.eu/strat-boardT-Pedia: http://urban.jrc.ec.europa.eu/t-pedia.

4 2016 CAPRI baseline was provided by the EC-JRC Directorate Sustainable Resource, Economics of Agriculture Unit (JRC.D.04).

5 NPV is a standard method used in capital budgeting to appraise long-term investments, by measuring discounted time series of expected cash inflows and outflows, while taking into account the time value of money.

6 The risk map of agricultural land abandonment represents the probability of occurrence. In terms of modelling is considered as suitability map and, however, partially responsible of the spatial allocation of future abandonment. The risk map covers the Eu and the UK.

7 See Table B1 (appendix B) for more detailed information.

8 The Farm Accountancy Data Network (FADN) is an instrument for evaluating the income of agricultural holdings and the impacts of the Common Agricultural Policy. The concept of the FADN was launched in 1965, when Council Regulation 79/65 established the legal basis for the organization of the network. It consists of an annual survey carried out by the Member States of the European Union. The services responsible in the Union for the operation of the FADN 3/94 collect every year accountancy data from a sample of the agricultural holdings in the European Union. Derived from national surveys, the FADN is the only source of microeconomic data that is harmonized, i.e. the bookkeeping principles are the same in all countries.

9 Farm Structure Survey (FSS) covers all agricultural holdings with an UAA of at least 1 ha or using market production as a threshold. The main purpose of FSS is to obtain reliable data, at regular timing intervals (two three years), on the structure of agricultural holdings in the European Union, in particular in land use, livestock and labour force. The first time conducted was in 1966–67. Approximately, every ten years, the FSS is conducted in the form of agricultural census, providing more detailed geographical levels. MSs transmit individual (micro) data to Eurostat, where they are stored in a database (Eurofarm). The legal basis for the FSS is regulation (EC) No1166/2008 of 19 November 2008.

10 Harmonization process consist mainly of 1) filling gaps: missing NUTS3 data are filled by scaling up data from sub-regions, extrapolation and interpolation according to the variable); 2) matching NUTS3 regions between FSS and FADN due to version discrepancies, specifically FSS used NUTS3 v.2006 and FADN used v.2003.

11 The biophysical factors receives the highest weight (0.4 out of 1) compared to the other two groups (0.3 out of 1 for each group).

12 LUCAS (Land use/cover area frame survey) provides and harmonized land use/cover statistics collected by Eurostat in cooperation with the Member States across the whole European Union's territory. In 2009, a soil module was included by the European Commission tended to construct a topsoil database based on samples carried out every three years by surveyors in the field (http://ec.europa.eu/eurostat/web/lucas/data). It should be noticed that in the LUCAS database, no abandonment is observed in Austria, Belgium, Denmark, Estonia, Croatia, Ireland, The Netherlands, Sweden and Slovenia.

13 Mountain areas have been spatially identified using the Less-Favoured Areas (LFA) classification map, corresponding to the class named “Totally mountain/hill areas” from the Spatial Dataset 2000–2006 based on GISCO Communes - Version 2.4.

14 With the highest absolute loss of more than 27 thousand ha expected in Terras de Trás-os-Montes (PT11E).

15 Nationwide, the largest absolute loss of about 33 thousand ha is, however, projected for Aveyron (FR622) in Southern France.

16 Projected about 48 thousand ha for Sassari (ITG25) and 35 thousand ha – for Nuoro (ITG26).

17 Korinthia (EL652) on the Peloponnese peninsula and the island of Lefkada (EL624).

18 Reported local shares of abandonment refers to the 35 study areas.

19 Abandonment hotspots numbered as 1, 2, 5, 8 and 10 covering the Alps, Pre-Alps and Central Pyrenees accounting for 79%, 97% and 84.1% of abandonment shares, respectively. Examples of local abandonment shares from LUISA: Montjustin (94%), Ourdon (90%), Aspin-Aure (90%), Grust (91%), Claix (93%), Séchilienne (88%), Saint-Sigismond (95%), Thônes (91%), Avignonet (79%), Fouillouse (81%), Jarjayes (86%), Chèze (77%), etc.

20 For the whole section, we represent in brackets the code number of the NUTS3 (region) or LAU2 (municipality) and the associated abandonment share.

21 Abandonment hotspots numbered as 12, 13, 14, 15, 16, 18, 19, 21, 22 and 23 are located across the Cantabrian Mountain, Central System, Sistema Betico, Central Pyrenees, Catalan Prelitoral and Iberian Range.

22 Abandonment hotspots 12, 13 and 22 with shares between 40 and 80% that correspond to the municipalities of: Allande, Teverga, Aller, Camaleño, Polaciones, Valdeolea and San Roque de Riomiera ranging from 38% to 53% of abandonment from LUISA.

23 Abandonment hotspots numbered as 14 and 15 with shares between 75% and 89% that correspond to the municipalities of: Puentes Viejas, Navarredonda y San Mamés, Lozoyuela, Manzanares el Real, San Ildefonso, Narrillos del Álamo, Orihuela, Medinilla, La Carrera and Piedrahita ranging from 42% to 81% shares from LUISA.

24 Abandonment hotspots numbered as 18 and 21 with shares between 40% and 71% that correspond to the municipalities of: Torre de Cabdella, Sort, Les Valls d'Aguilar, Prullans, La Vansa i Fórnols, El Pont de Suert, Valderrobres, Monroyo, Castellote and Villarluengo ranging from 31% to 85%.

25 Abandonment hotspots numbered as 19 and 20 with shares between 42% and 99% that correspond to the municipalities of: San Asensio, Cenicero, Sotés, Haro, Tarazona, Borja, Ainzón, Fuendejalón and Morata de Jalón ranging from 33% to 81% of abandonment from LUISA.

26 Abandonment hotspots numbered as 16 and 23 with shares between 36% and 70% that correspond to the municipalities of: Villaluenga del Rosario, Ronda, Alpujarra de la Sierra, Nevada, Albondón, Torvizcón, Fiñana, Fondón, Felix, Lubrín, Macael, Oria and Chirivel ranging from 45% to 74% of abandonment from LUISA.

27 Abandonment hotspots numbered as 6, 9, 32 and 36 with shares between 18% and 33% that correspond to the municipalities of: Skierbieszów, Krzczonów, Leśniowice, Krynice, Rybnik, Lyski, Godów, Proszowice, Koniusza and Łapanów ranging from 31% to 64% of abandonment from LUISA.

28 Abandonment hotspots numbered as 26, 27 and 28 with 11.7%, 40% and 26.6% shares, respectively, that correspond to the municipalities of: Sondalo, Sernio, Valdidentro, Tirano, Colorina, Berbenno di Valtellina, Baselga di Pinè, Pergine Valsugana, Vigolo Vattaro, Sant'Orsola Terme, Bedollo, Terlago, Micigliano, Borona, Antrodoco, Petrella ranging from 8% to 67% in Italy from LUISA outcomes; the Austrian municipalities in the point 27 and 11 are: Tux, Gerlos, Krimml, Absam, Gshnitz, Zirl and Vals ranging from 21% to 61% of abandonment from LUISA.

29 The abandonment hotspot numbered as 7 has a 20% share of local abandonment that corresponds to the municipalities in: Jakubova Voľa, Ďurková, Drienica, Fričovce, Rokycany, Vyšný Medzev, Medzev, Háj and Bôrka ranging from 25% to 95% of abandonment from LUISA.

30 There is not any studied abandonment area (hotspot) for Lithuania reported from the consulted literature.

31 Abandonment hotspots numbered as 33(4), 34(5) and 35(6) with shares between 10.1% and 50% that correspond to the municipalities of: Viimsi, Ikšķiles novads, Salaspils novads and Cesvaines novads ranging from 11% to 40% of abandonment from LUISA. Haapsalu and Surju have the highest shares, 40% and 28%, respectively.

32 Abandonment hotspots numbered as 29 and 30 with shares between 20% and 77% in Greece correspond to the Greek island municipalities where the highest abandonment shares ranging from 4 to 5% up to 52% (with the maximum values in Νισύρου (8119) and Κέας (Ιουλίδος) (8209) accounting for 52% and 37% respectively) though most of the shares are below 10%.

33 Abandonment hotspots numbered as 24 and 25 (Portugal) with abandonment shares of 40% and 77%, respectively, correspond to the municipalities where the highest abandonment shares ranging from 6% up to 34% though most of the shares are below 5%.

34 Only LUCAS (Land use/cover area frame survey, 2015 version) covers some Member States with roughly 389 observations. This is the information that we have used for the second strategy of validation

35 In the surroundings of Lisbon and Oporto where some municipalities show the highest abandoned shares ranging from 56% to 85%, as well as near the National Park of Peneda with abandonment shares around 50%.

36 In the southern and northwestern part of Latvia can be found some municipalities that show the highest abandoned shares near 15% such as Aknīstes novads (0560800), Rojas novads (0888301) or Mērsraga novads (0887600).

37 As Alcantara et al. (2013) identified, LUISA is able to find in Lithuania (LT00A – 2%; LT009 – 5%). Especially in the municipalities such as Antakalnio seniūnija (1301), Panerių seniūnija (1309), Vidiškių seniūnija (4504), Salako seniūnija (4348), etc. where the highest abandonment shares range from 10% to 26%.

38 In Olsztyński (PL622-11%) and Szczecinecko-pyrzycki (PL427–9.5%) some municipalities show abandoned shares ranging from 37% to 50%.

39 In Nizke Tatry Žilinský kraj. (SK031-10%) some municipalities show abandoned shares ranging from 25% to 95%.

40 Especially in Harghita (RO12 - 7%), Mures (RO125–6.3%) or Sibiu (RO126–4.3%) regions

41 Kuemmerle et al. (2008), Keenleyside and Tucker (2010), Kozak et al. (2004) and Pointereau et al. (2008) in Poland; Muller and Kummerle et al. (2009) in Romania; Peterson and Aunap (1998), Nikodemus et al. (2005), Ruskule et al. (2013) and Van Dijk et al. (2005) in Baltics countries; Pinto Correia (1993) in Portugal; Gomez-Moreno (1989), Alonso-Sarría et al. (2016) and Perpiña Castillo et al. (2020) in the Southeastern part of Spain.
